# Supplementary material for: From TDP-43/RNA complex formation to disease-linked TDP-43 aggregation through a structural and cellular approach
Source: Nat Commun. 2026 Jan 21;17:1631. doi: 10.1038/s41467-026-68346-y (PMC12905282; doi:10.1038/s41467-026-68346-y)
Supplement: Supplementary file 1 — Supplementary Information [file 41467_2026_68346_MOESM1_ESM.pdf]

SUPPLEMENTARY INFORMATION:

Supplementary Figures and Legends (Page 2 – 44)

Supplementary Table 1 for pET plasmids for protein production and purification (Page 45)

Supplementary Table 2 for SAXS data (Page 46 - 49)

Supplementary Table 3 for sequences of the oligonucleotides used for EMSA (Page 50)

Supplementary Table 4 for ITC data (Page 51)

SUPPLEMENTARY FIGURE LEGENDS:

TDP-43 (a.a., 1-277)	WT	E17R	R52E
Molecular mass determination predicted by SAXS ($MM_{correclation\ volume}$ [kDa])	45	34	34.5
$MM_{sequence}$ [kDa] : 31.5			

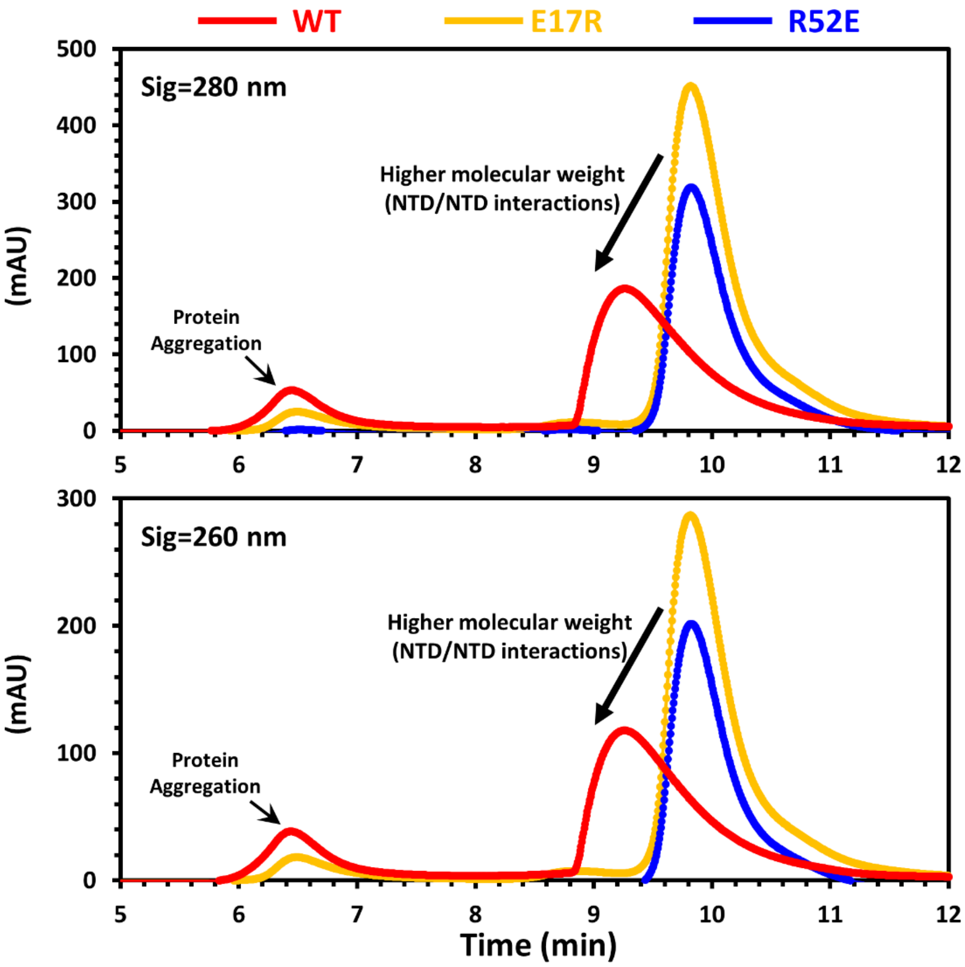

Supplementary Figure 1: The single mutant (R52E or E17R) are sufficient to prevent NTD/NTD interactions of TDP-43

Estimated molecular weight (MM) from data SAXS and UV absorption elution profiles of mutant E17R, mutant R52E, and wild type TDP-43 (a.a., 1-277). The measurements of NTD-RRM1-2 fragments (a.a., 1-277) were performed by using a Water BioResolve2.5-200 column. The upper panel displays absorbance measured at 280 nm, while the lower panel displays absorbance at 260 nm. We noticed a significant decrease of the elution time certainly due to an increase in the size of wild type TDP-43, but not R52E or E17R.

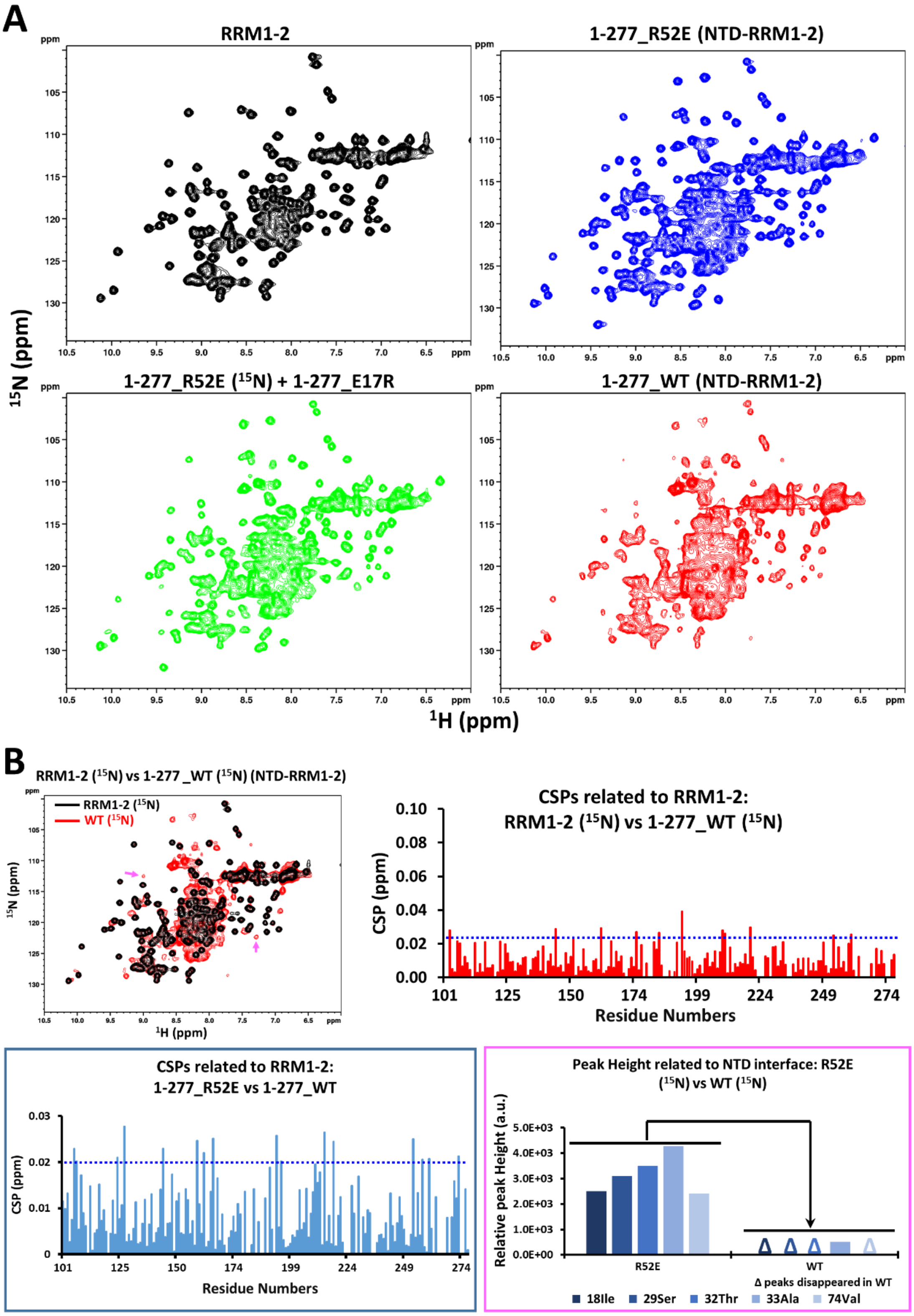

Supplementary Figure 2: The presence of NTD or NTD/NTD interactions do not affects RRM1-2 residues

A) NMR spectra of ^{15}N-labelled RRM1-2 (a.a., 101-277), wild type TDP-43, R52E (a.a., 1-277), and R52E in the presence of E17R (R52E+E17R). ^{15}N-labelled R52E initiates NTD/NTD interactions in the presence of unlabeled E17R.

B) Upper left panel: Superimposed NMR spectra of ^{15}N-labelled RRM1-2 and wild type TDP-43. Upper right panel: CSPs of indicated residues when comparing RRM1-2 residues. Blue dotted line shows three times the standard deviation of the CSP values. Residues without a bar were not assigned or overlapped in 2D spectra. Lower left panel: CSPs between wild type and R52E. Blue dotted line shows three times the standard deviation of the CSP values. NTD/NTD interactions does not lead to significant CSPs in residues located in the RRM1-2 domain. Lower right panel: Relative peak heights of NTD residues with or without NTD/NTD interactions. NMR peaks of these residues are indicated by pink arrows in 2D spectra.

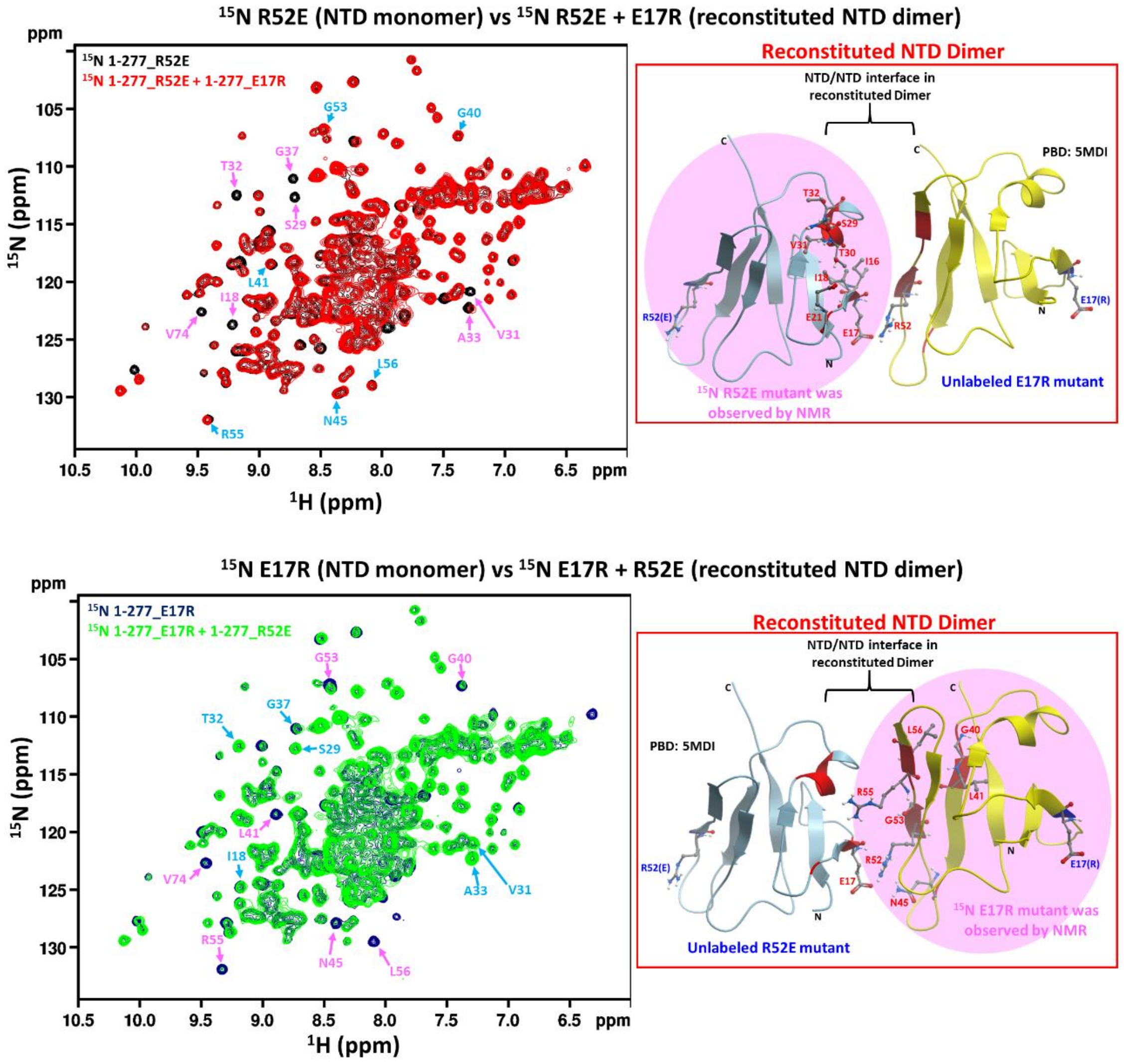

Supplementary Figure 3: NTD dimers reconstituted by E17R and R52E mutants

Left upper panel: Superimposed NMR spectra of 30µM ^{15}N-labeled R52E in the absence or presence of 30µM unlabeled E17R. NTDs in two mutants form the reconstituted dimer via non-mutated side (peaks disappeared or broaden, pink arrows) but not through the mutated side (peaks stable, blue arrows).

Left lower panel: Superimposed NMR spectra of 30µM ^{15}N-labeled E17R in the absence or presence of 30µM unlabeled R52E. NTDs in two mutants form the reconstituted dimer via non-mutated side (peaks disappeared or broaden, pink arrows) but not through the mutated side (peaks stable, blue arrows).

Right panel: Schematic view on NTD/NTD interactions based on structural data. ^{15}N-labeled protein observed by NMR is in the pink circle.

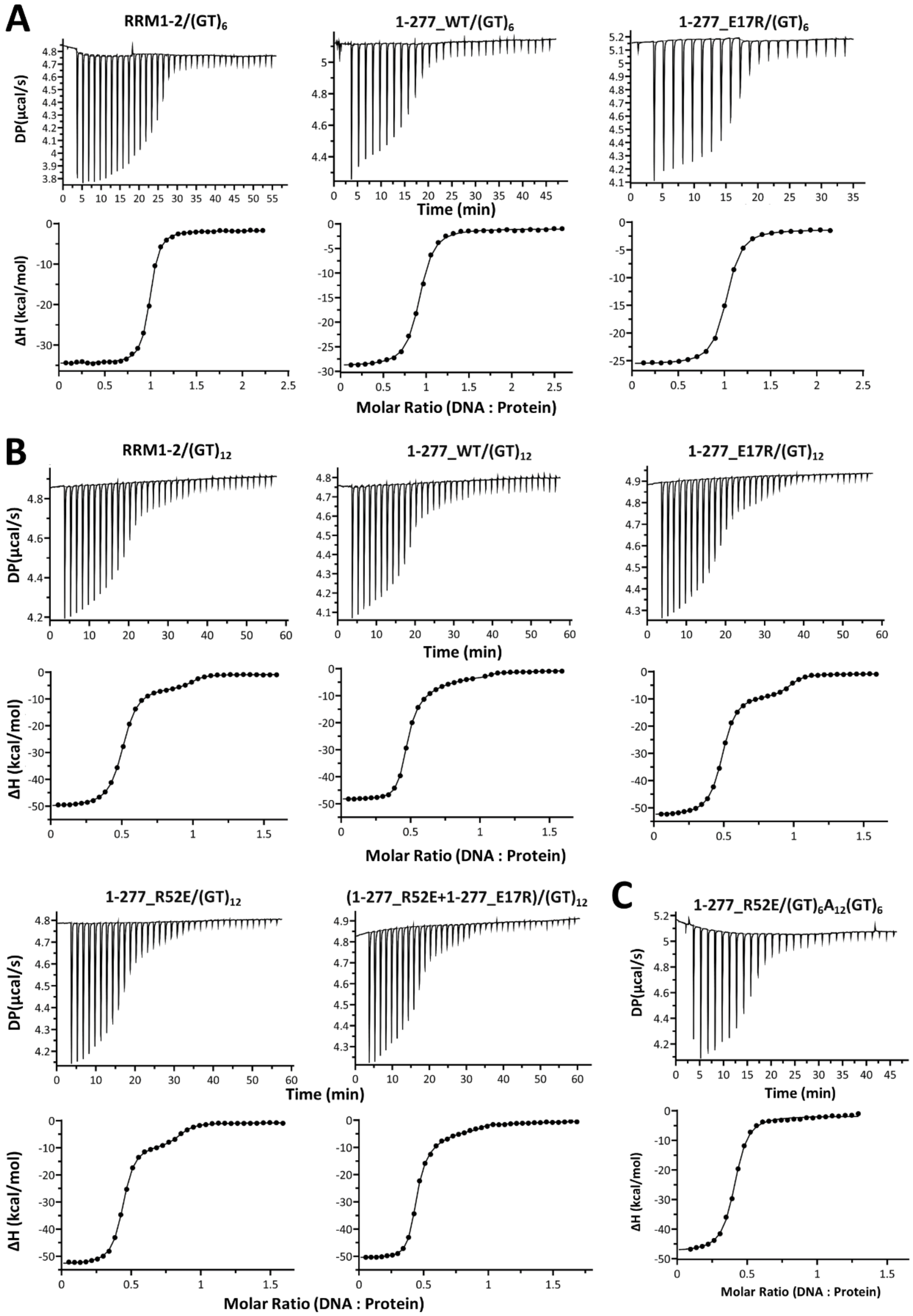

Supplementary Figure 4: ITC curves showing the negative impact of NTD/NTD interaction of TDP-43 on cooperative association to (GT)$_{12}$

A), B) and C) ITC data obtained with indicated TDP-43 constructs and DNA oligonucleotides. Thermodynamic parameters and ITC statistics are shown in Supplementary Table 4.

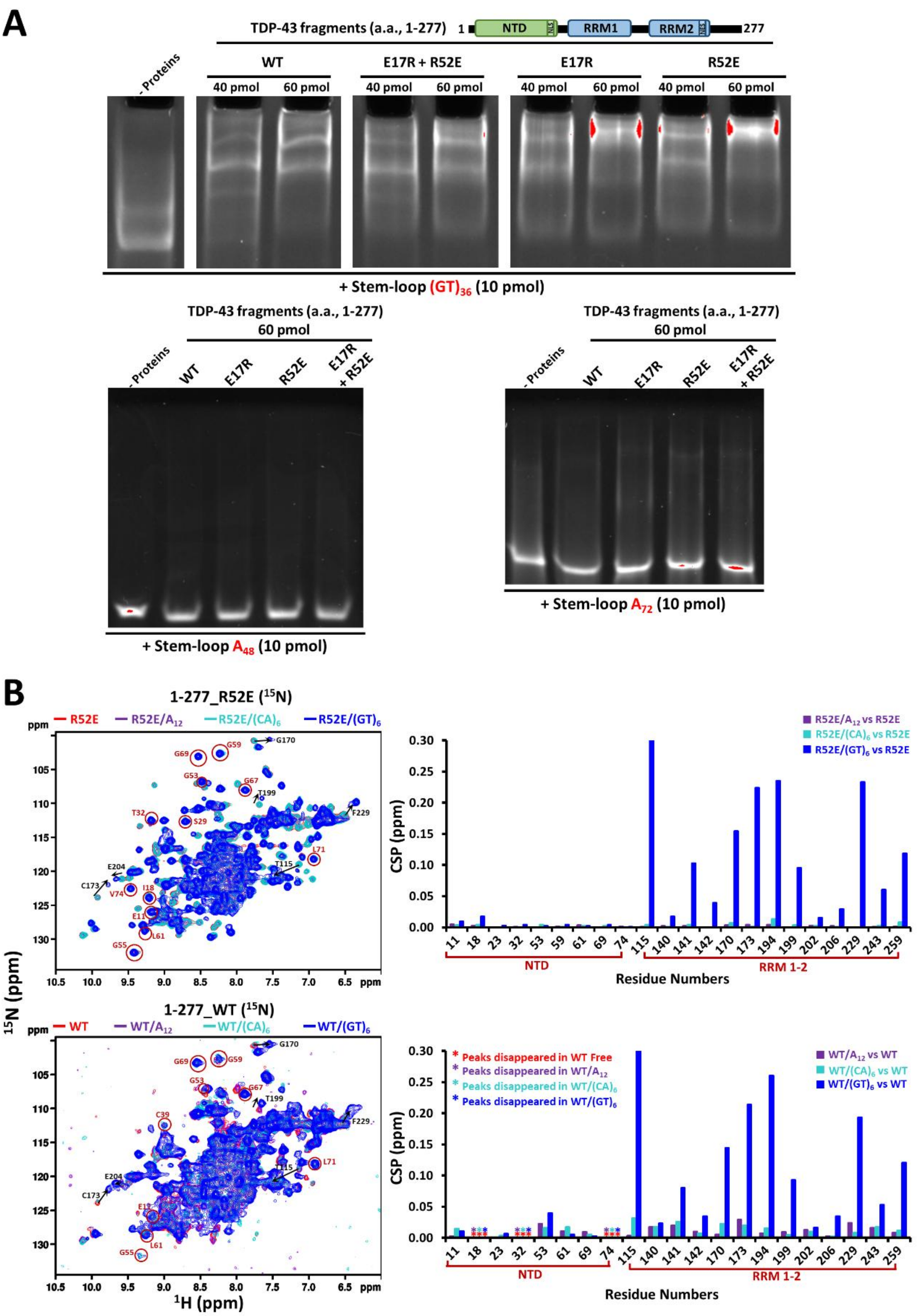

Supplementary Figure 5: TDP-43 fails to bind to poly-A or CA repeats oligonucleotides

A) EMSA revealed that wild type TDP-43, E17R mutant, R52E mutant and the reconstituted dimer E17R+R52E (TDP-43 fragments, a.a., 1-277) do not interact with stem-loop A_{48} or stem-loop A_{72} but interact with stem-loop $(GT)_{36}$ oligonucleotides.

B) NMR spectra revealed that no significant shift of peaks of NTD residues in complex with A_{12} or $(CA)_6$, comparing to TDP-43 alone.

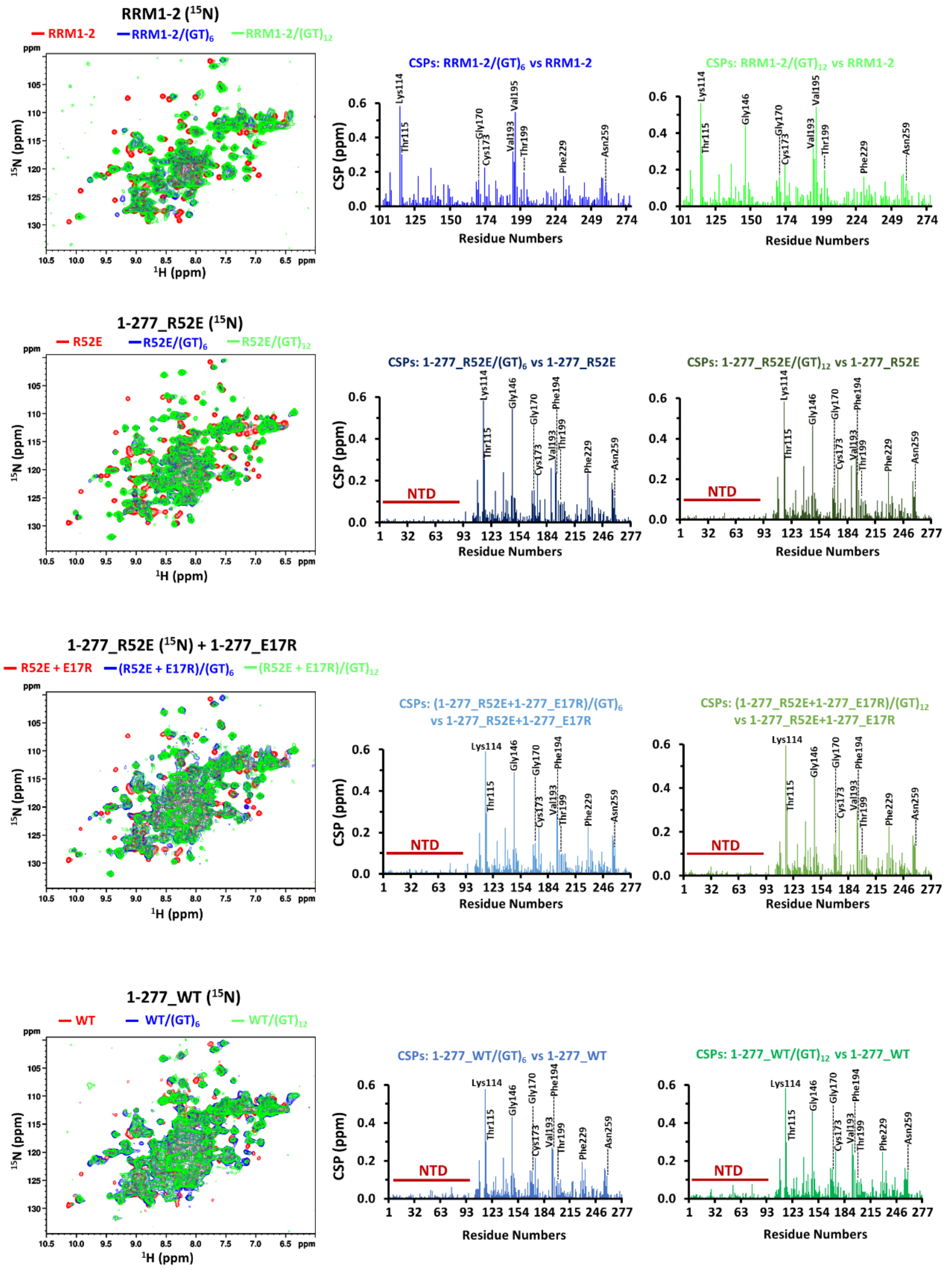

Supplementary Figure 6: NTD/NTD interactions do not interfere with the binding of RRM1-2 residues to nucleic acids

Left panels: Superimposed NMR spectra of different proteins in the presence or absence of GT repeats DNA oligonucleotides: ^{15}N-labelled RRM1-2 (a.a., 101-277), wild type TDP-43, R52E (a.a., 1-277), and R52E in the presence of unlabeled E17R (R52E+E17R, a.a.,1-277). Protein

alone (red), protein in the presence of $(GT)_6$ oligonucleotides (blue) and protein in the presence of $(GT)_{12}$ oligonucleotides (green). ^{15}N-labelled R52E initiates NTD/NTD interactions in the presence of unlabeled E17R.

Right panels: CSPs show the difference between protein in the presence of $(GT)_6$ or $(GT)_{12}$ and protein alone. Significant CSPs are observed only in binding residues from RRM1-2 (assigned in black). Compared to RRM1-2, no additional CSPs show in wild type TDP-43, R52E and R52E+E17R. Residues without a bar were not assigned or overlapped in 2D spectra.

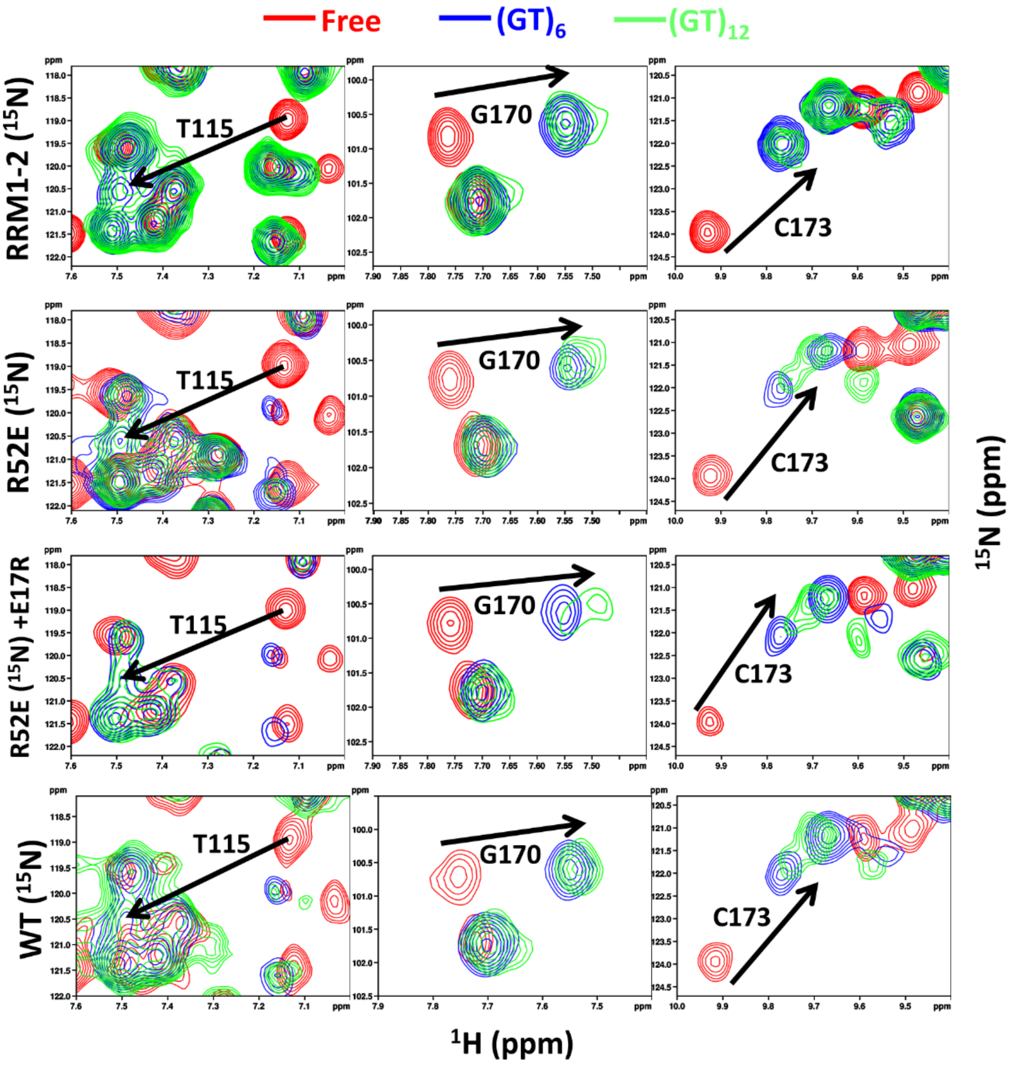

Supplementary Figure 7: Zoomed-in view on the chemical shift perturbations for TDP-43 residues in RRMs in the presence or absence of indicated oligonucleotides

NTD/NTD interactions are possible in wild type TDP-43 and R52E+E17R but absent in R52E alone or RRM1-2. We noted limited interferences of NTD/NTD interactions on CSPs associated to the interactions of RRM1-2 with DNA.

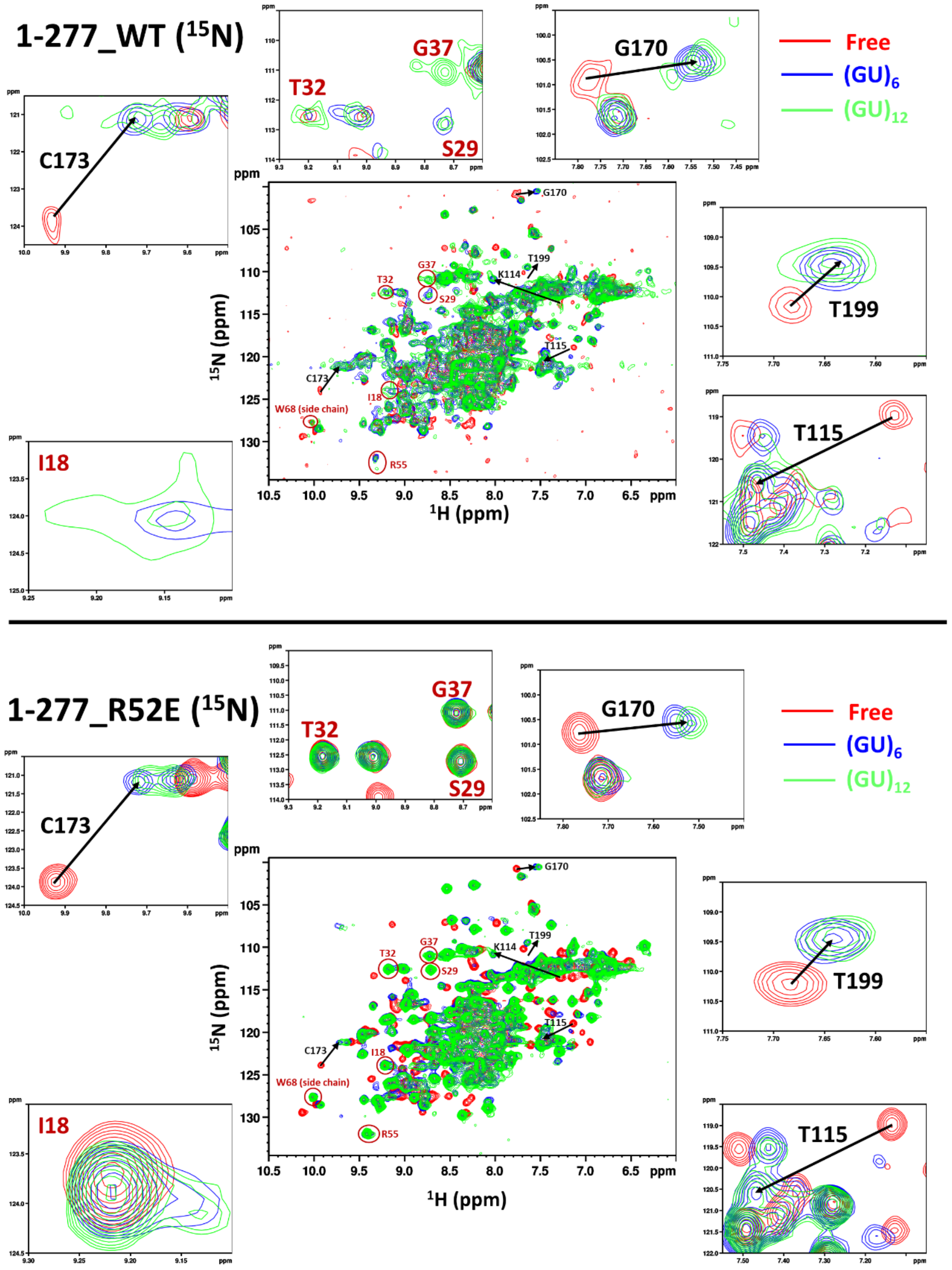

Supplementary Figure 8: NMR spectra of wild type TDP-43 or R52E in the presence of GU RNA repeats oligonucleotides

Red circle indicates NTD residues. Arrows show the CSPs of RRM1-2 consecutive to the interaction with $(GU)_6$ and $(GU)_{12}$. $(GU)_{12}$ leads to the appearance of NMR peaks of NTD residues with wild type TDP-43 as compared with $(GU)_6$.

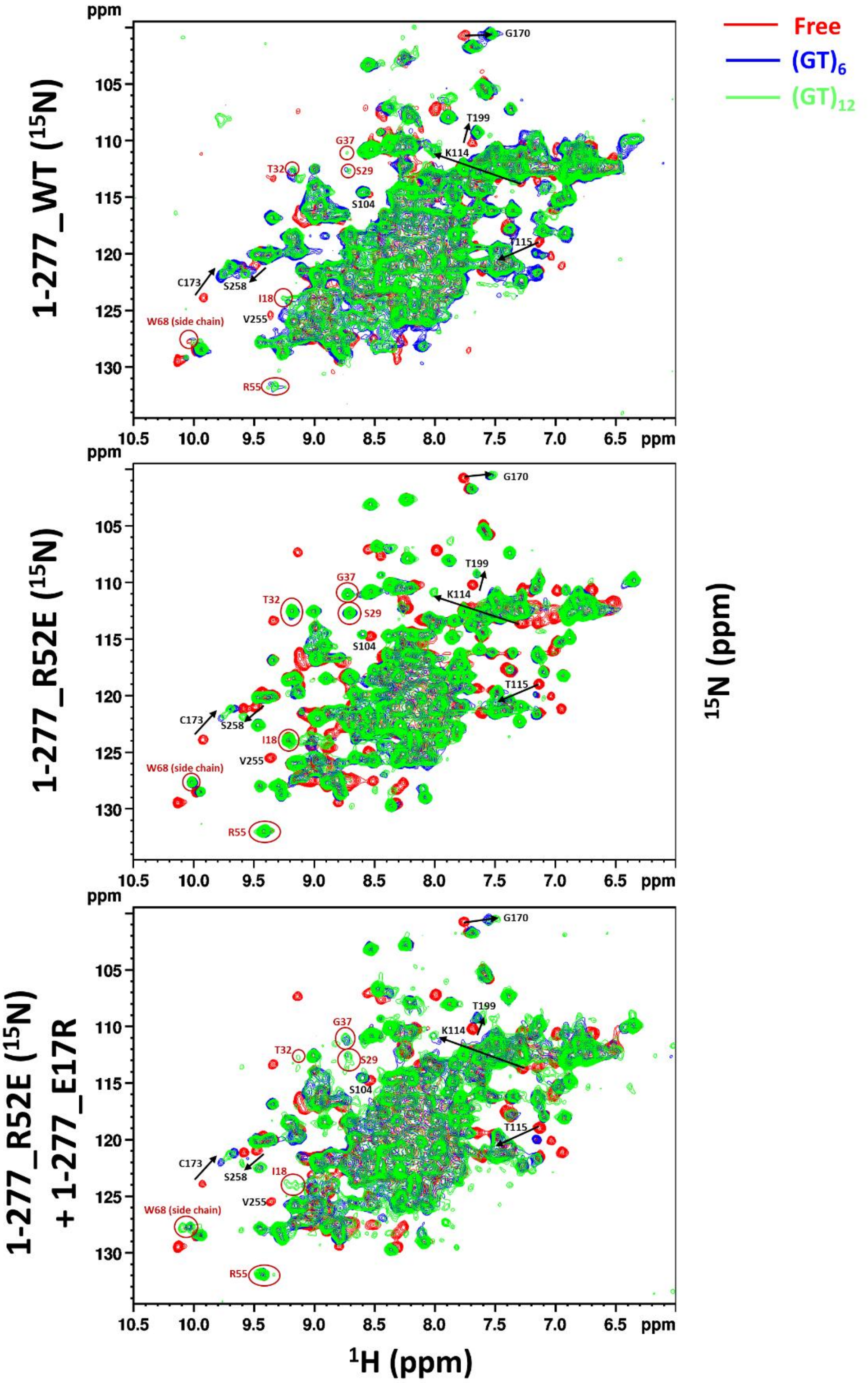

Supplementary Figure 9: NMR Spectra of indicated TDP-43 constructs in the presence or absence of $(GT)_6$ or $(GT)_{12}$ oligonucleotides

Red circle indicates NTD residues. Arrows show the CSPs of RRM1-2 consecutive to the interaction with $(GT)_6$ and $(GT)_{12}$.

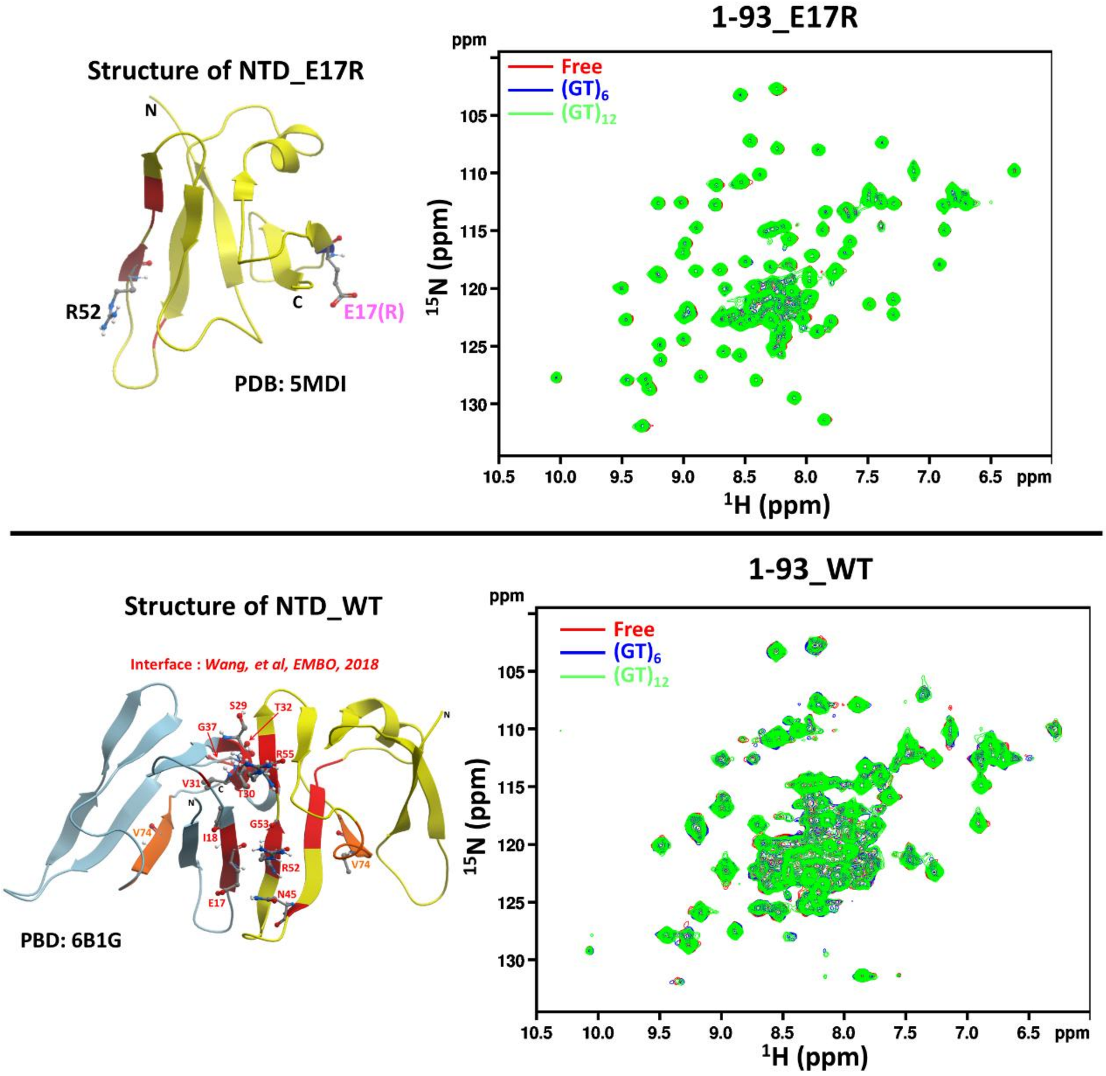

Supplementary Figure 10: NMR spectra of NTD fragments (a.a., 1-93)

E17R NTD does not initiate NTD/NTD interactions and does not interact with $(GT)_6$ and $(GT)_{12}$. WT NTD initiates NTD/NTD interactions leading to the disappearance of multiple peaks of residues located in the NTD/NTD interface. NTD/NTD interactions are not affected by the presence of $(GT)_6$ or $(GT)_{12}$.

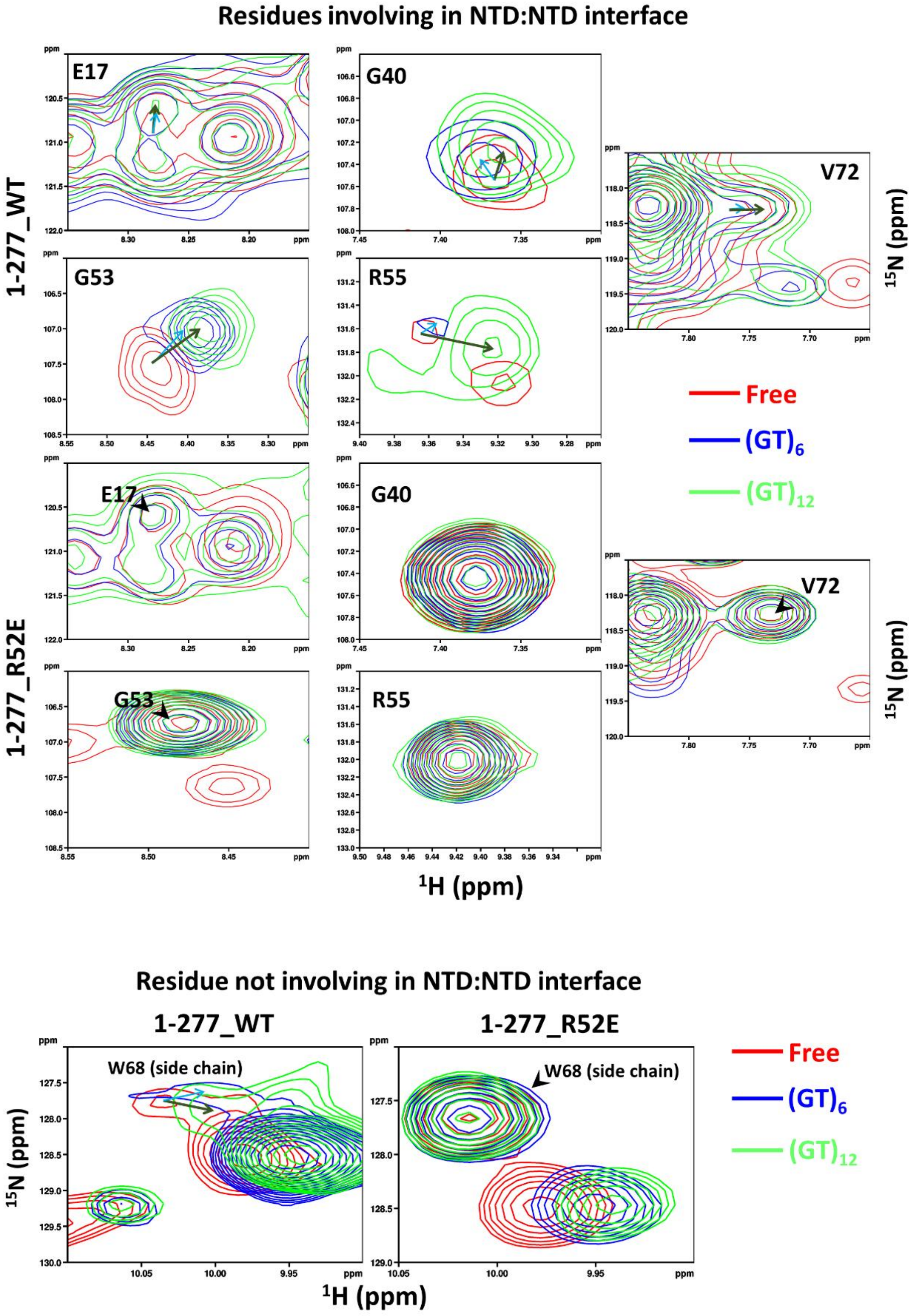

Supplementary Figure 11: Examples of large CSPs observed with wild type TDP-43 but not with R52E mutant in the presence of $(GT)_{12}$

Upper panels: Large CSPs displayed by residues involved in NTD:NTD interface, are observed in the case of wild type TDP-43 in the presence of $(GT)_{12}$ but not with R52E mutant in the presence of $(GT)_{12}$.

Lower panels: Large CSPs of the side chain of W68, absent in NTD:NTD interface, is also observed in the case of wild type TDP-43 in the presence of $(GT)_{12}$ but not with R52E mutant in the presence of $(GT)_{12}$.

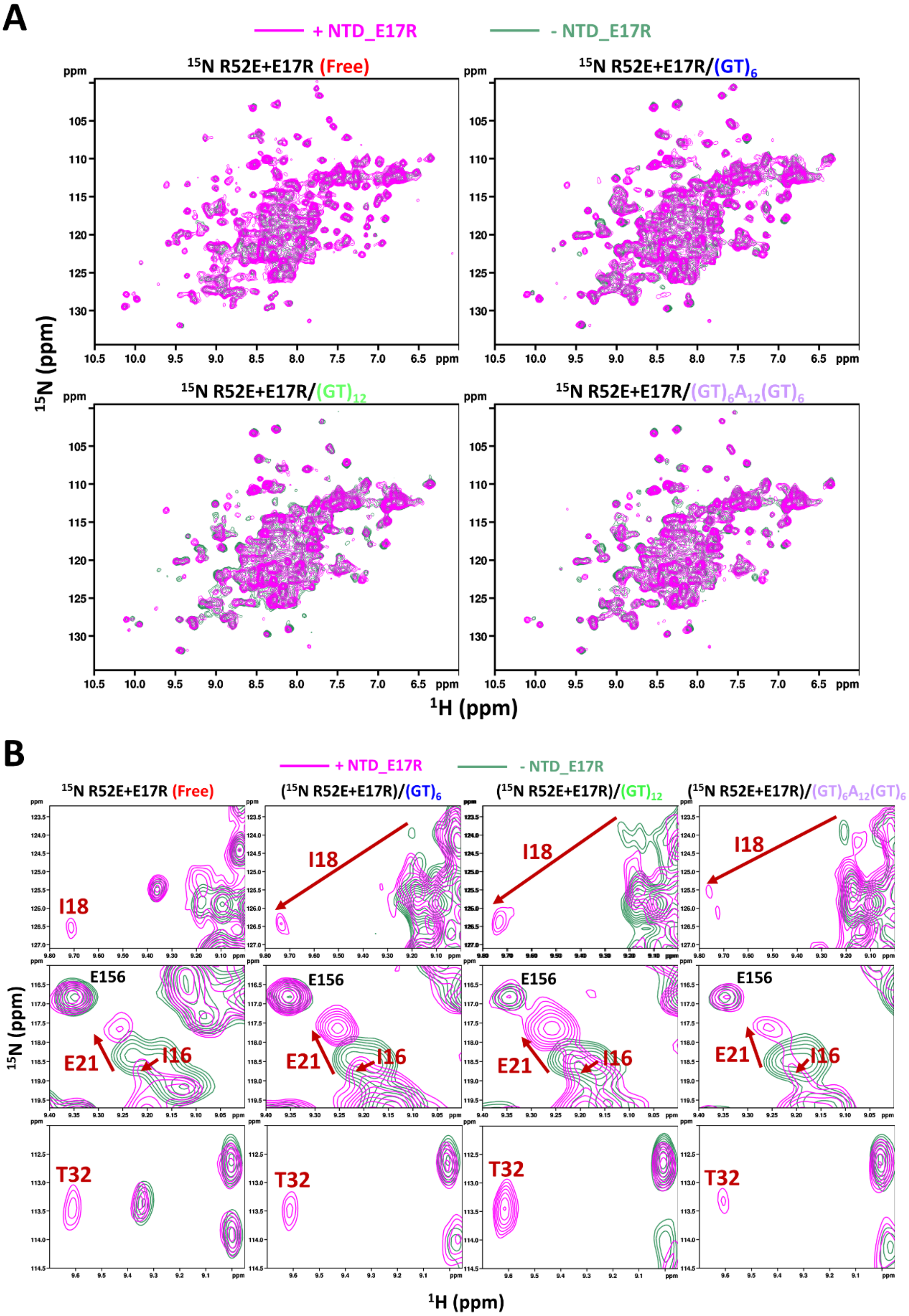

Supplementary Figure 12: NMR spectra of reconstituted NTD/NTD interaction between ^{15}N-labelled R52E (a.a., 1-277) and unlabeled E17R (a.a., 1-277) at equimolar concentration in the presence or absence of E17R NTD (a.a., 1-93) in large excess

A) NMR spectra obtained under indicated conditions

B) Zoom in on the NMR spectra showing the reappearance of NTD residues in the presence of E17R NTD in excess. This phenomenon is more pronounced in the presence of $(GT)_{12}$ than in the presence of $(GT)_6$ or $(GT)_6A_{12}(GT)_6$, which indicate that a cooperative association to oligonucleotide antagonizes NTD/NTD interactions.

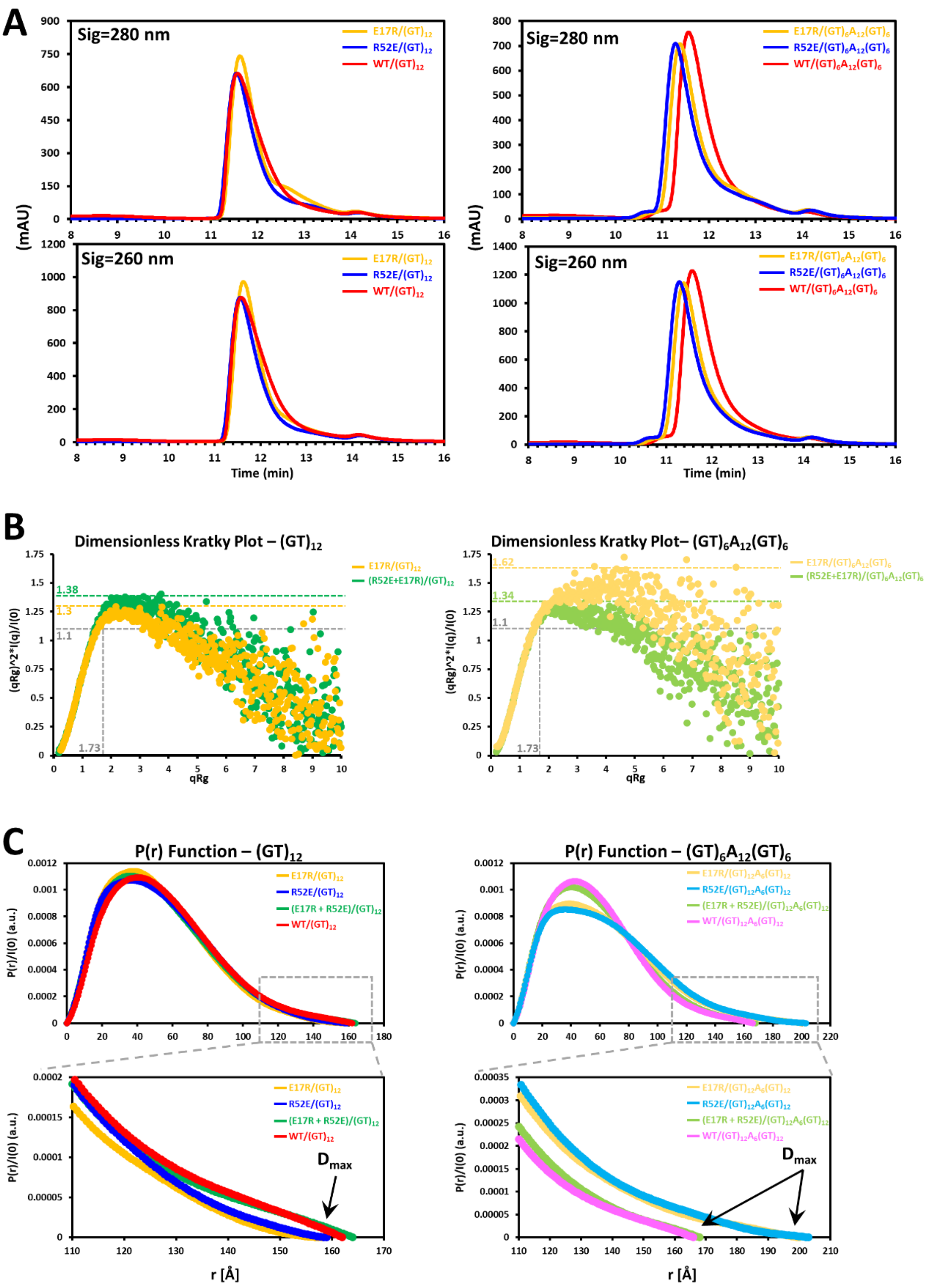

Supplementary Figure 13: Size exclusion chromatography and SAXS of TDP-43 in complex with DNA oligonucleotides

A) UV absorption elution profiles of E17R, R52E, and wild type TDP-43 (a.a., 1-277) in complex with indicated oligonucleotides. The upper panel displays absorbance measured at 280 nm, while the lower panel displays absorbance at 260 nm. We noticed a significant

increase of the elution time certainly due to a decrease in the size of the complex when wild type TDP-43, but not R52E or E17R, interact with $(GT)_6A_{12}(GT)_6$.

B) Dimensionless Kratky Plots of E17R and E17R+R52E in interactions with $(GT)_{12}$ or $(GT)_6A_{12}(GT)_6$. E17R+R52E, but not E17R alone, reduced the sizes of the complexes in the presence of $(GT)_6A_{12}(GT)_6$.

C) P(r) function plots of wild type TDP-43, E17R, R52E and E17R+R52E in interactions with $(GT)_{12}$ or $(GT)_6A_{12}(GT)_6$. Black arrows show the D_{max} value of the complex.

A

Samples	Rg [Å]	D_{max} [Å]
WT/(GU)$_{12}$	41.71±0.46	163
(R52E+E17R) /(GU)$_{12}$	40.54±0.24	159
R52E/(GU)$_{12}$	41.17±0.38	161
E17R/(GU)$_{12}$	42.01±0.16	159

Samples	Rg [Å]	D_{max} [Å]
WT/(GU)$_6$A$_{12}$(GU)$_6$	39.65±0.35	151
(R52E+E17R) /(GU)$_6$A$_{12}$(GU)$_6$	41.11±0.24	156
R52E/(GU)$_6$A$_{12}$(GU)$_6$	48.26±0.32	198
E17R/(GU)$_6$A$_{12}$(GU)$_6$	45.62±0.32	195

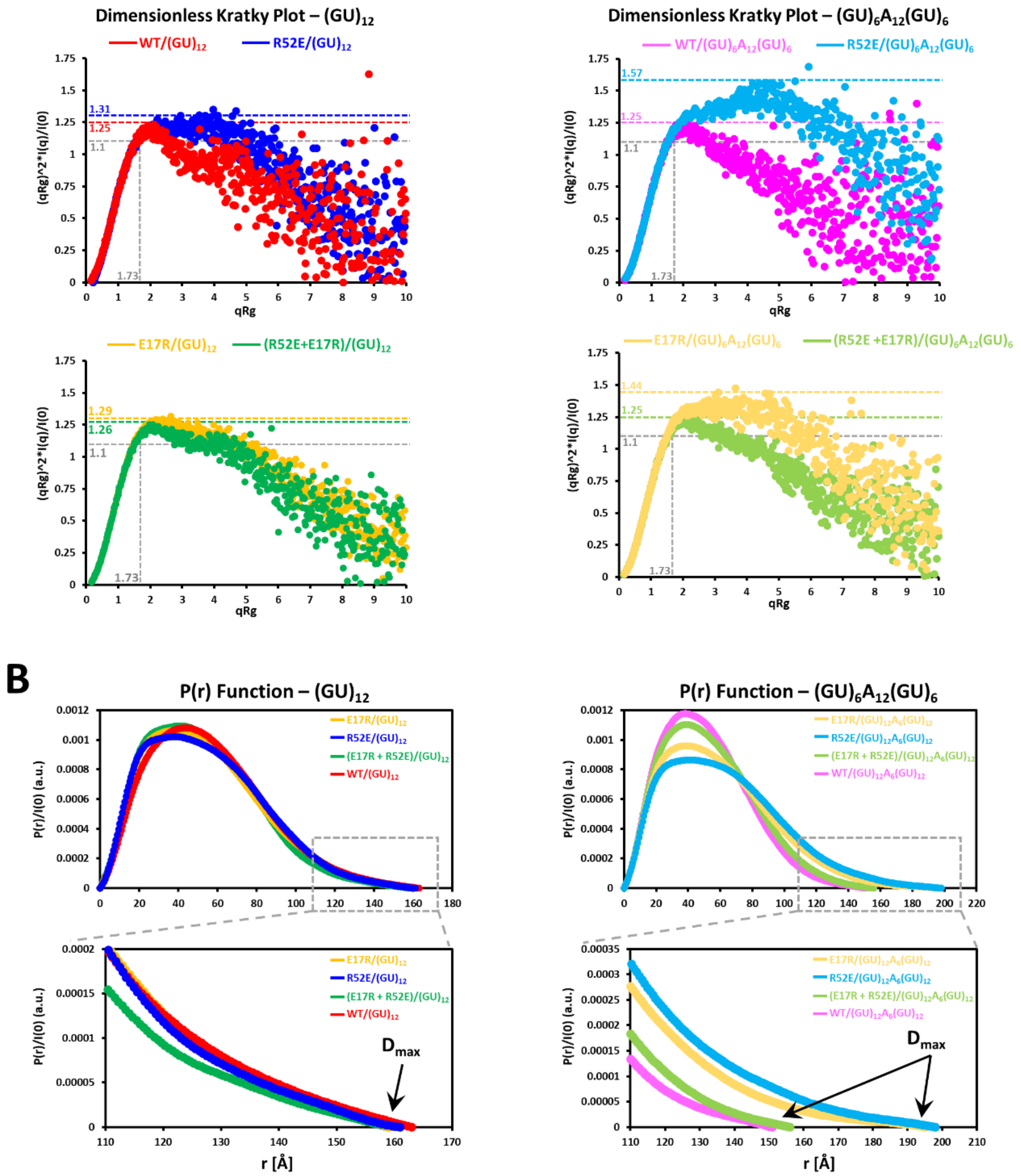

Supplementary Figure 14: SAXS of TDP-43 in complex with RNA oligonucleotides

A) Upper tables: R_g and D_{max} values obtained from SAXS analysis with the indicated conditions. As for RNA oligonucleotides, we noticed a significant decrease in the size of the complex when wild type TDP-43, but not R52E or E17R, interacts with (GU)$_6$A$_{12}$(GU)$_6$. Lower panels: Dimensionless Kratky Plots of wild type TDP-43, R52E, E17R and E17R+R52E in interactions with (GU)$_{12}$ or (GU)$_6$A$_{12}$(GU)$_6$. E17R+R52E and wild type TDP-43, but not R52E or E17R, reduced the size of the complexes in the presence of (GU)$_6$A$_{12}$(GU)$_6$.

B) P(r) function plots of wild type TDP-43, E17R, R52E and E17R+R52E in interactions with $(GU)_{12}$ or $(GU)_6A_{12}(GU)_6$. Black arrows show the D_{max} value of the complex.

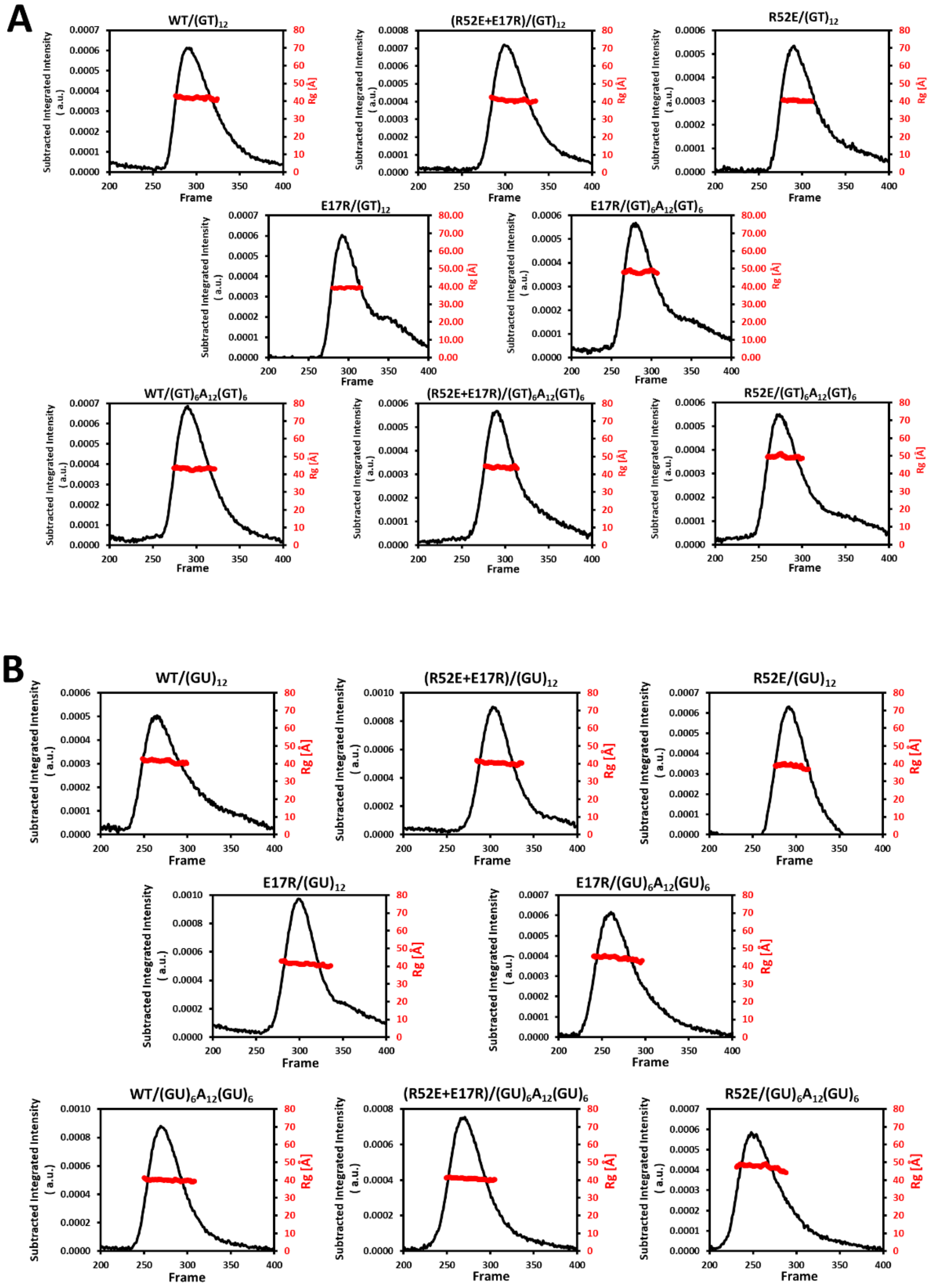

Supplementary Figure 15: SAXS elution profiles of TDP-43 in complex with DNA or RNA oligonucleotides

A) SAXS elution profiles indicate, for each protein complex with DNA oligonucleotides, the evolution of subtracted integrated intensity (black curve) and Rg values (Radius of Gyration, red dots), related to the experimental values presented in the plots of Figure

3A and B. The monodispersity of the complex is observed indicating the stable evolution of the Rg values. The shoulder corresponds to an excess of free proteins.

B) SAXS elution profiles indicate, for each protein complex with RNA oligonucleotides, the evolution of subtracted integrated intensity (black curve) and Rg values (Radius of Gyration, red dots), related to the experimental values presented in the tables in Supplementary Figure 14, Panel A). The shoulder corresponds to an excess of free proteins.

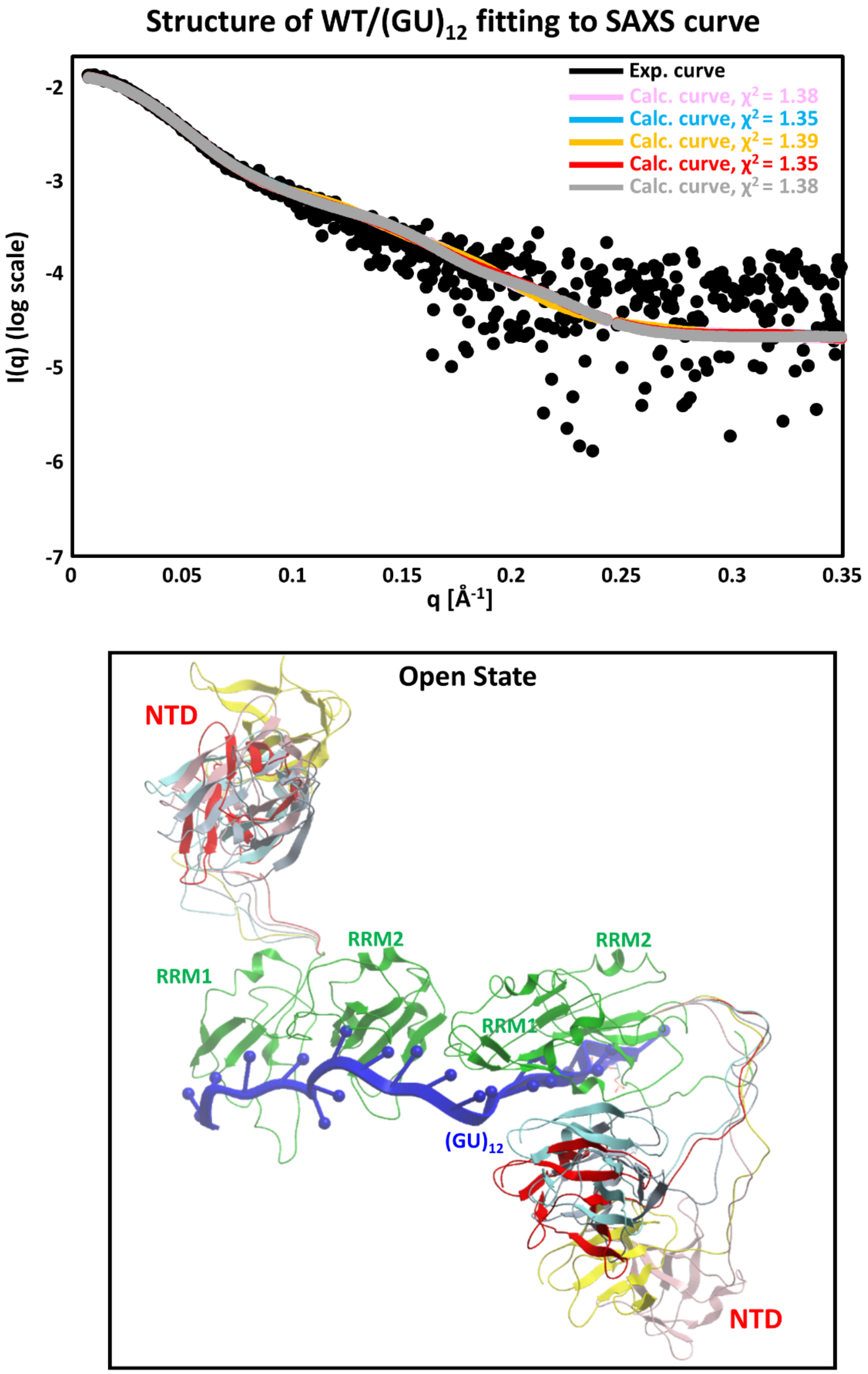

Supplementary Figure 16: DADIMODO model of two TDP-43 units in complex with $(GU)_{12}$

Upper panel: Superimposition of calculated from best-fit DADIMODO models (colored curves) and experimental (black dots) SAXS curves corresponding to wild type TDP-43 (a.a., 1-277) bound to $(GU)_{12}$. The corresponding χ^2 values are indicated.

Lower panel: Superimposition of structures of the TDP-43 complex with $(GU)_{12}$ obtained and adjusted based on SAXS data using DADIMODO, consistent with SAXS curves. Note the large separation distance between the NTDs, which does not allow their interaction, leaving the NTDs free to interact with NTDs of other TDP-43 proteins (long-ranged interaction with other GU sequences, notably).

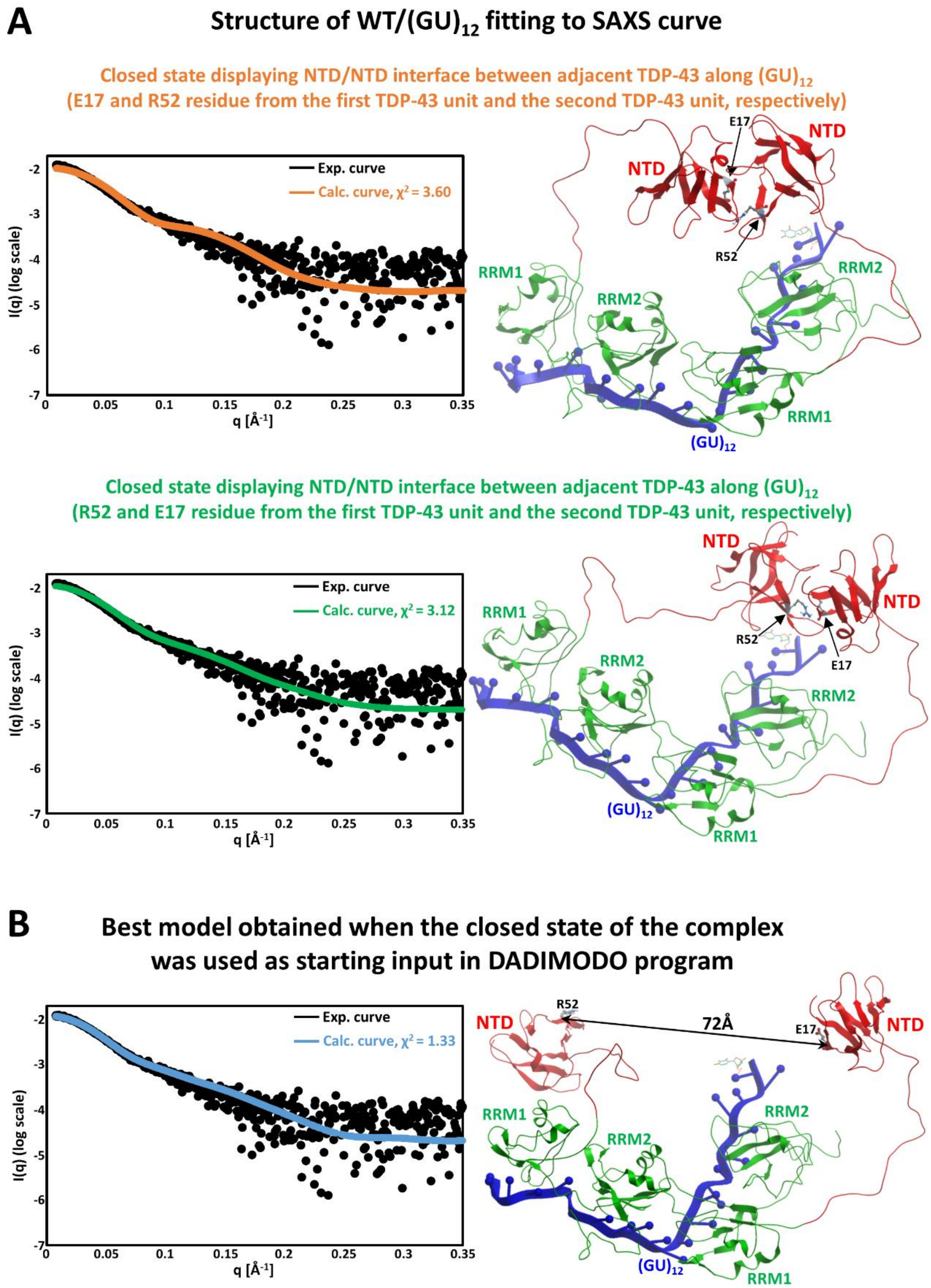

Supplementary Figure 17: The experimental SAXS curve is not consistent with the closed-state models of two TDP-43 units in complex with $(GU)_{12}$

A) Two closed models of two TDP-43 units in complex with $(GU)_{12}$ were generated by DADIMODO program. We applied distance constraints (4-5 Å) between R52 residue from NTD of one TDP-43 unit, and E17 residue from NTD of the second TDP-43 unit.

The best-fitting models are linked to significantly elevated χ^2 values, 3.60 (upper panel) or 3.12 (lower panel) when E17 (from the first unit) interacts with R52 (from the second unit) or vice versa (R52/E17), respectively.

B) The best-constrained model ($\chi^2 = 3.12$) was subsequently used as the starting point for a new calculation performed without any distance constraints by using DADIMODO program. In this case, the best-fitting model obtained with a χ^2 value of 1.33 similar to the initial calculations (Supplementary Figure 16), corresponds to an open model in which the two NTD domains have moved apart by approximately 72 Å.

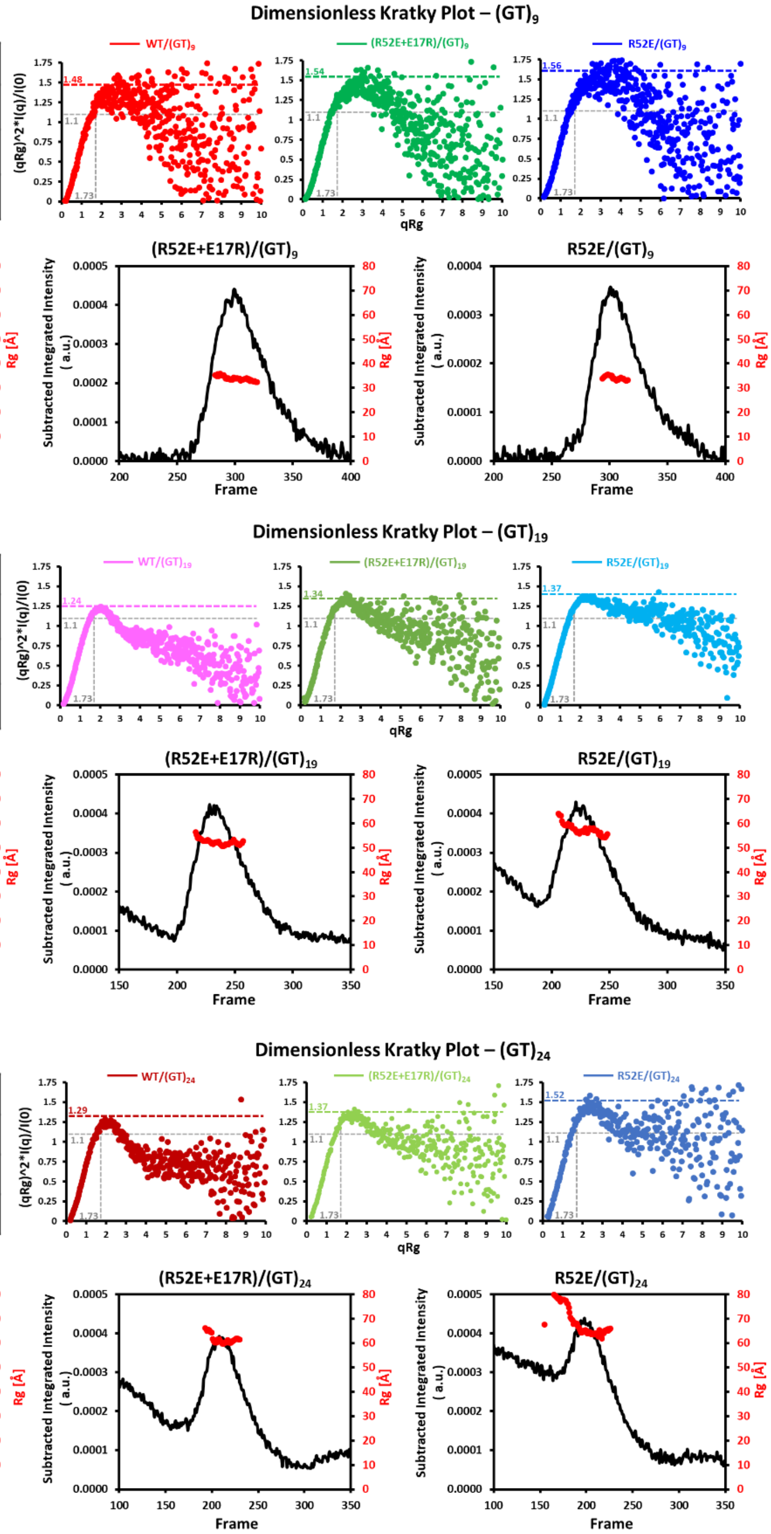

A

Samples	Rg [Å]	D_{max} [Å]
WT/(GT)$_9$	37.70±0.91	139
(R52E+E17R) /(GT)$_9$	35.10±0.48	134
R52E/(GT)$_9$	35.79±0.59	132

B

Samples	Rg [Å]	D_{max} [Å]
WT/(GT)$_{19}$	47.35±0.17	201
(R52E+E17R) /(GT)$_{19}$	52.67±0.63	225
R52E/(GT)$_{19}$	56.84±0.25	248

C

Samples	Rg [Å]	D_{max} [Å]
WT/(GT)$_{24}$	55.44±0.72	209
(R52E+E17R) /(GT)$_{24}$	61.26±0.83	231
R52E/(GT)$_{24}$	67.64±1.43	300

Supplementary Figure 18: SAXS of TDP-43 in complex with GT oligonucleotides of varying lengths

A), B) and C) Upper left table: R_g and D_{max} values obtained from SAXS analysis with wild type, R52E or R52E+E17R (a.a., 1-277) in presence of the indicated number of GT repeats. Upper right panel: Dimensionless Kratky Plots of wild type, R52E or R52E+E17R in complex

with indicated number of GT repeat oligonucleotides. Lower panels: SAXS elution profiles indicate, for each protein complex, the evolution of subtracted integrated intensity (black curve) and R_g values (red dots), related to the experimental values presented in upper left tables. We noticed a significant increase in the size of the complex with the length of the oligonucleotide target in the case of R52E protein but to a lesser extent in the case of equimolar mix R52E+E17R or wild type TDP-43. The shoulders at frame 100 are higher oligomeric structures, which have been separated by using the REGALS algorithm available in RAW.

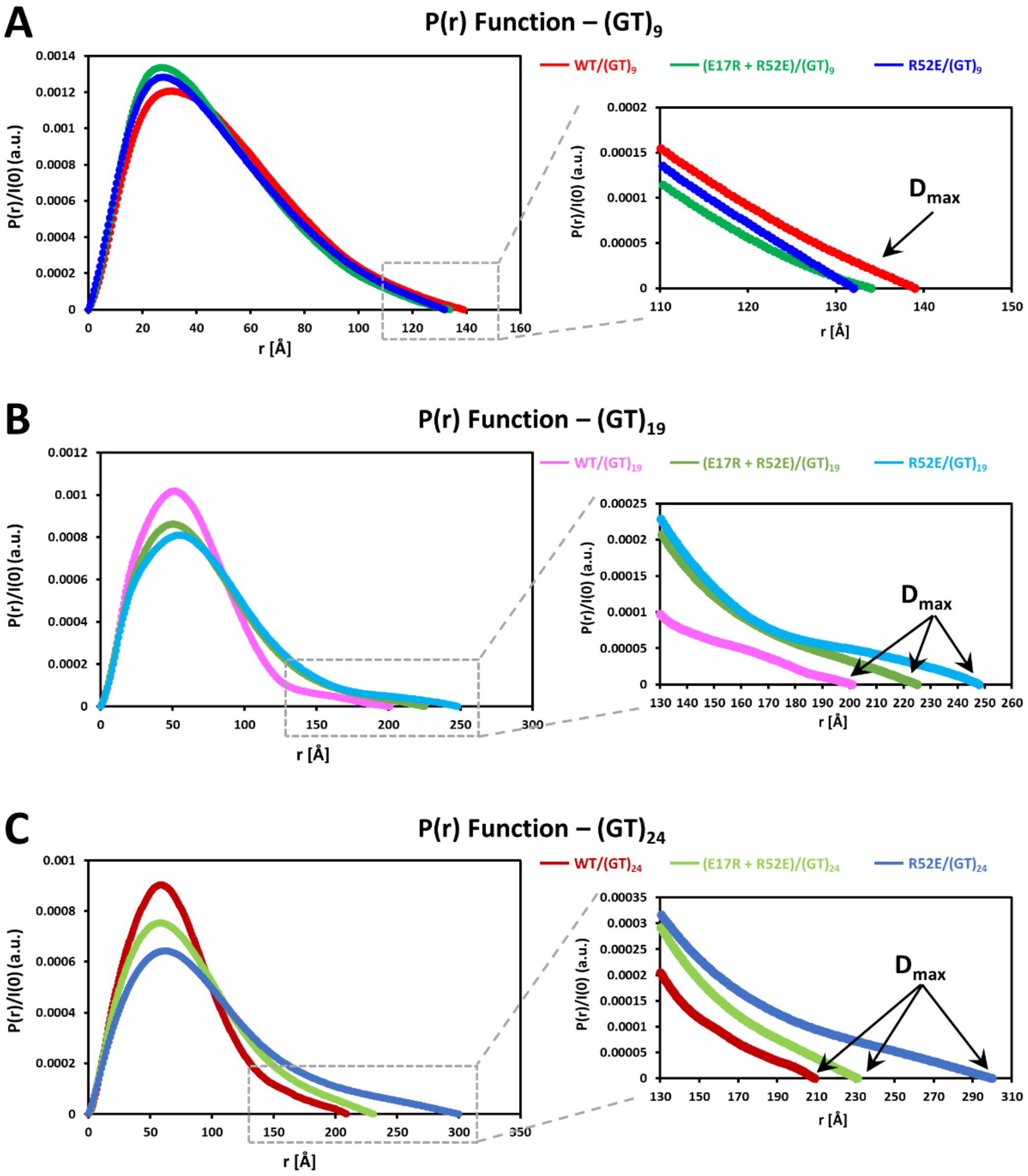

Supplementary Figure 19: P(r) function plots of TDP-43 in complex with GT oligonucleotides of varying lengths

A), B) and C) P(r) function plots of wild type TDP-43, R52E and E17R+R52E in complex with indicated number of GT repeats oligonucleotides. Black arrow indicates the D_{max} value of the complex.

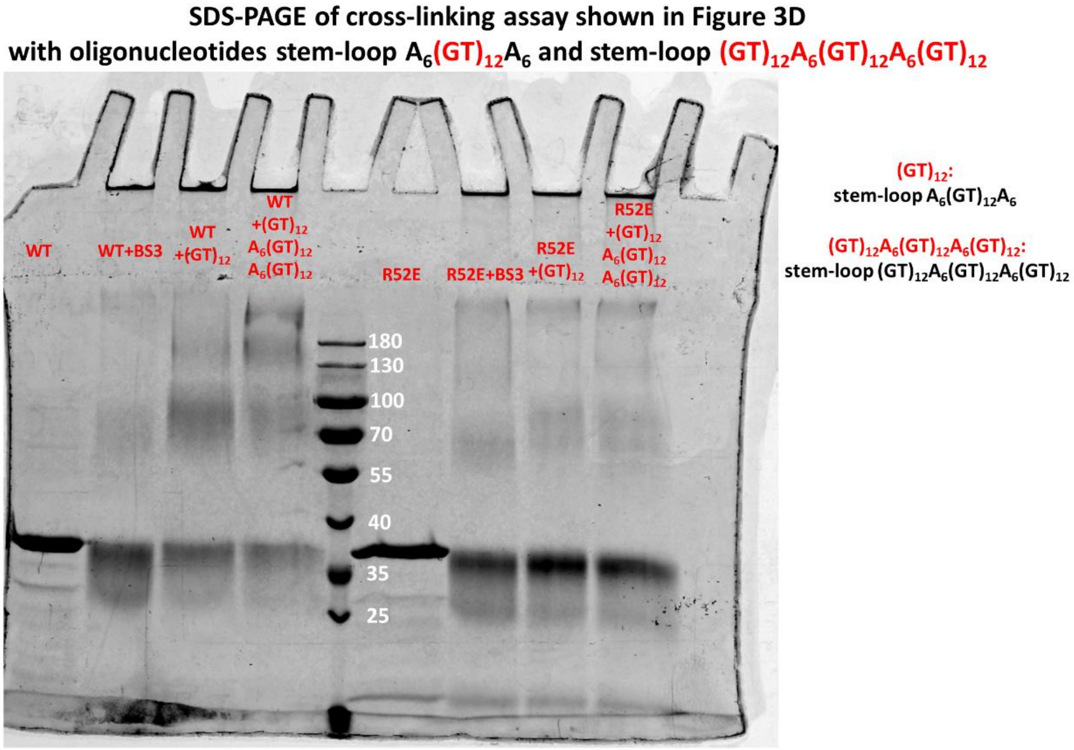

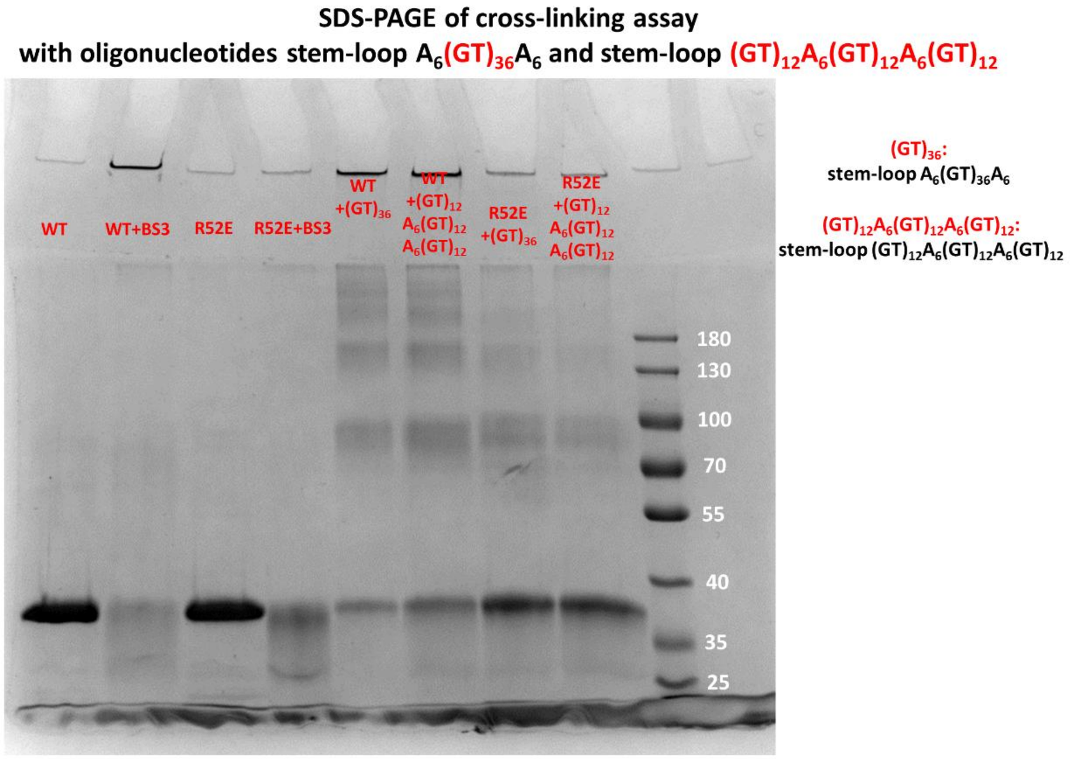

Supplementary Figure 20: Cross-linking assays

Upper panel: Protein cross-linking assay showing the formation of TDP-43 multimers (a.a., 1-277) in the presence of stem-loop $(GT)_{12}A_6(GT)_{12}A_6(GT)_{12}$ but not $A_6(GT)_{12}A_6$ with wild type TDP-43 (data shown in Figure 3D).

Lower panel: Similar experiments in the presence of $(GT)_{12}A_6(GT)_{12}A6(GT)_{12}$ oligonucleotide and an extended GT-repeat loop, $A_6(GT)_{36}A_6$ oligonucleotide. Again, $(GT)_{12}A_6(GT)_{12}A_6(GT)_{12}$ is able to initiate the formation of protein multimers in the case of wild type TDP-43 but not R52E mutant. Of note, we notice the appearance of higher-order assembly of wild type TDP-43 in presence of $A_6(GT)_{36}A_6$ because the oligonucleotide is sufficiently long to bend on itself as observed for longest oligonucleotides in SAXS data (Figure 3C).

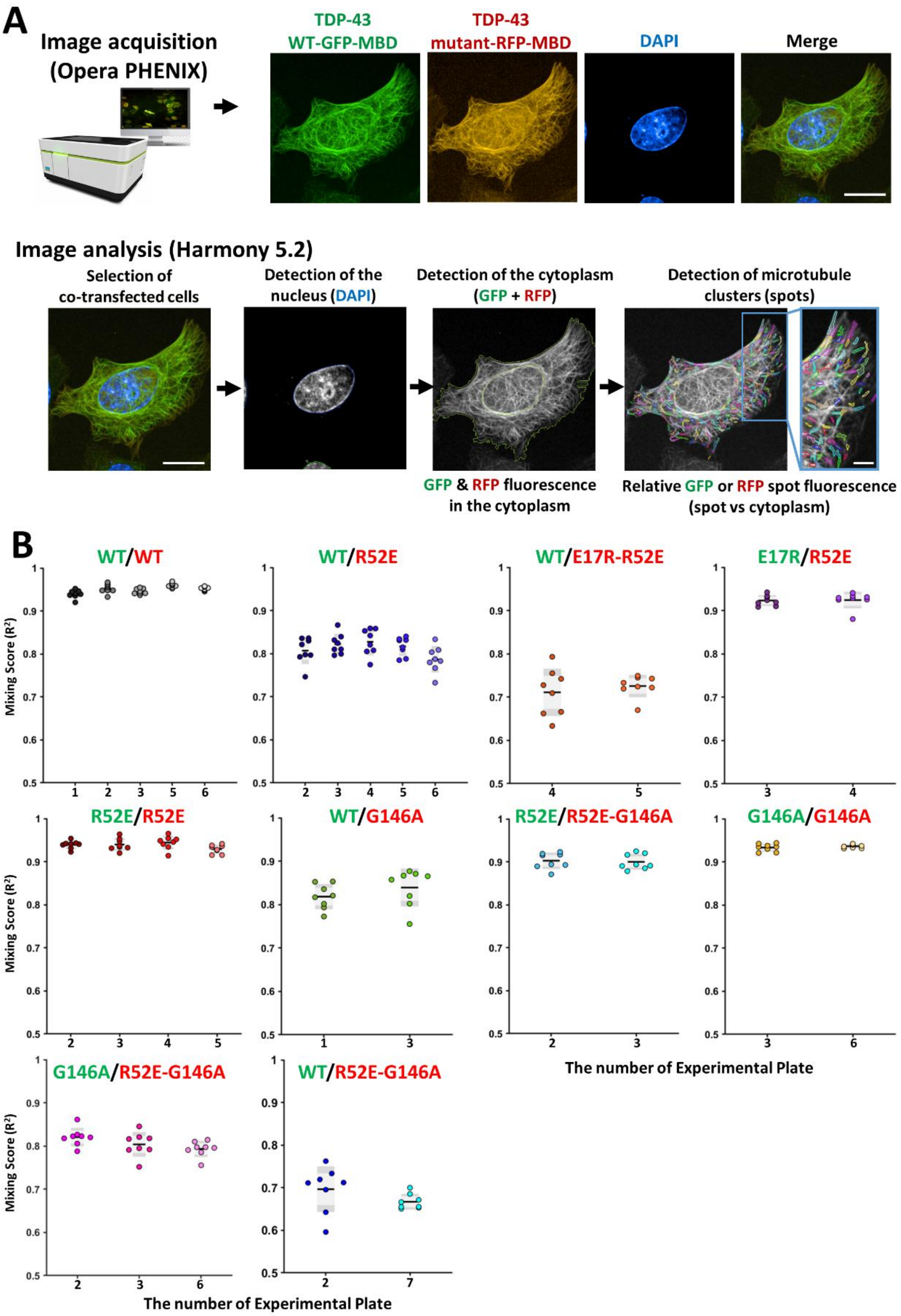

Supplementary Figure 21: Image processing leading to the automated detection of microtubule spots along microtubules

A) Images are taken in an automated manner with a HCS imager operating with water immersed lenses to benefit from a higher resolution. 96-well plates were used for all the cellular data presented. DAPI signal was used to detect the nuclei (Harmony software

function). Both RFP and GFP signals were used to detect elongated spot along microtubules (Harmony software function). RFP and GFP fluorescence were measured for each plot and used to measure the mixing score between RFP- and GFP-labeled RBPs. Scale bar: 20µm. Zoom scale bar: 5 µm.

B) The experimental replicates for different samples in MT bench assays. One dot represents the mean value in one well (n=8 wells).

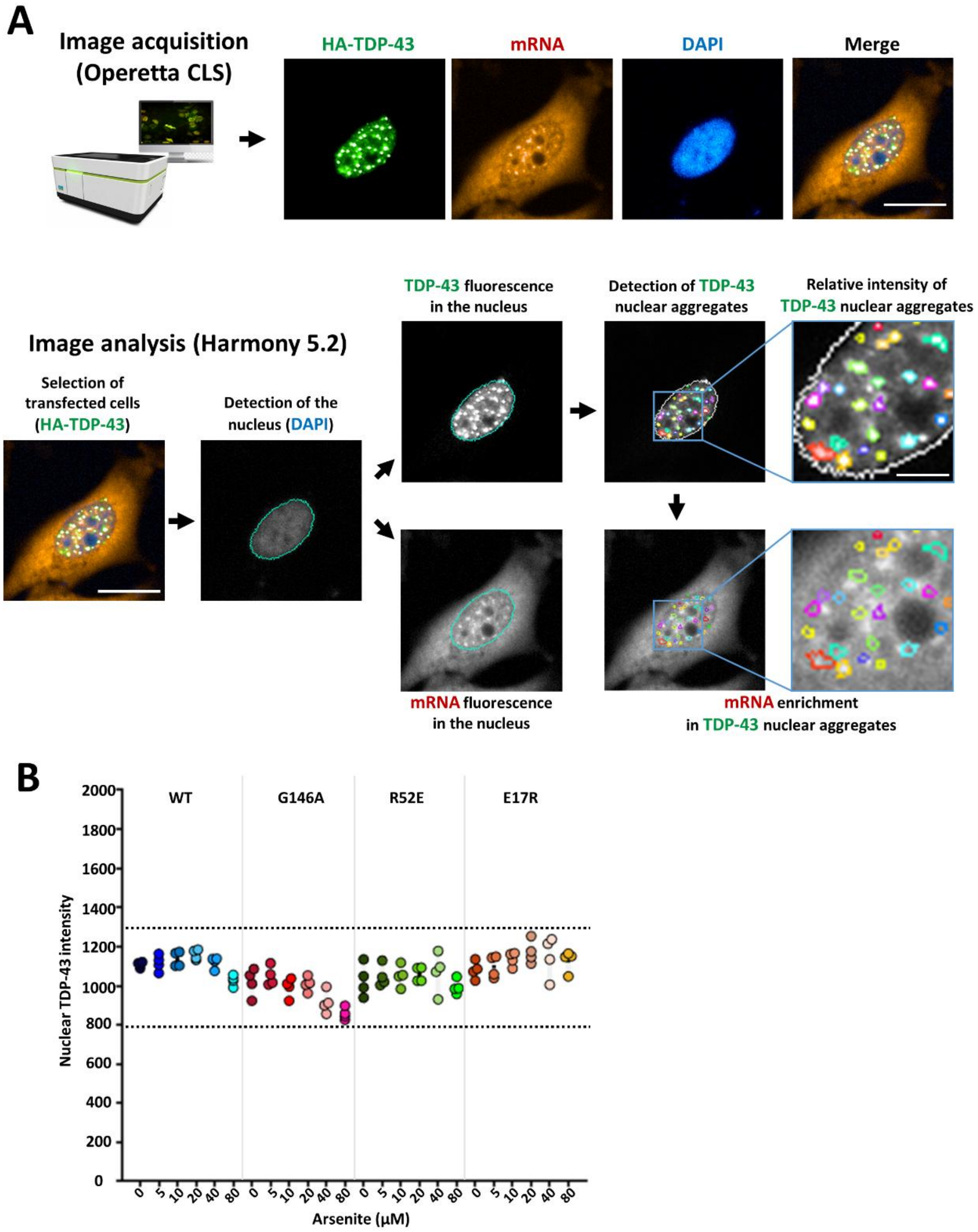

Supplementary Figure 22: Image processing leading to the automated detection of nuclear TDP-43-rich aggregates

A) Images are taken in an automated manner with a HCS imager. DAPI signal was used to detect the nuclei (Harmony software function). HA-antibody was used to detect HA-labeled TDP-43-rich aggregates in the nuclei of HeLa cells (Harmony software function). Simultaneously, mRNA was detected by in situ hybridization with Cy3-labbelled poly-dT probes. Scale bar: 20µm. Zoom scale bar: 5 µm.

B) Mean nuclear TDP-43 intensity obtained from cells expressing HA-TDP-43 or indicated mutants. Hela cells were exposed to indicate arsenite concentrations for 1 H prior to

fixation. The background value has been set to zero. The measurements indicate a similar HA expression for all the conditions tested, especially without arsenite. However, we can notice a slight decreased in TDP-43 nuclear intensity for the highest arsenite concentrations with wild type TDP-43 and more markedly with G146A, but not with R52E or E17R, which may correlate with the appearance of TDP-43 aggregates, especially with G146A. One dot represents the mean value in one well (n=8 wells).

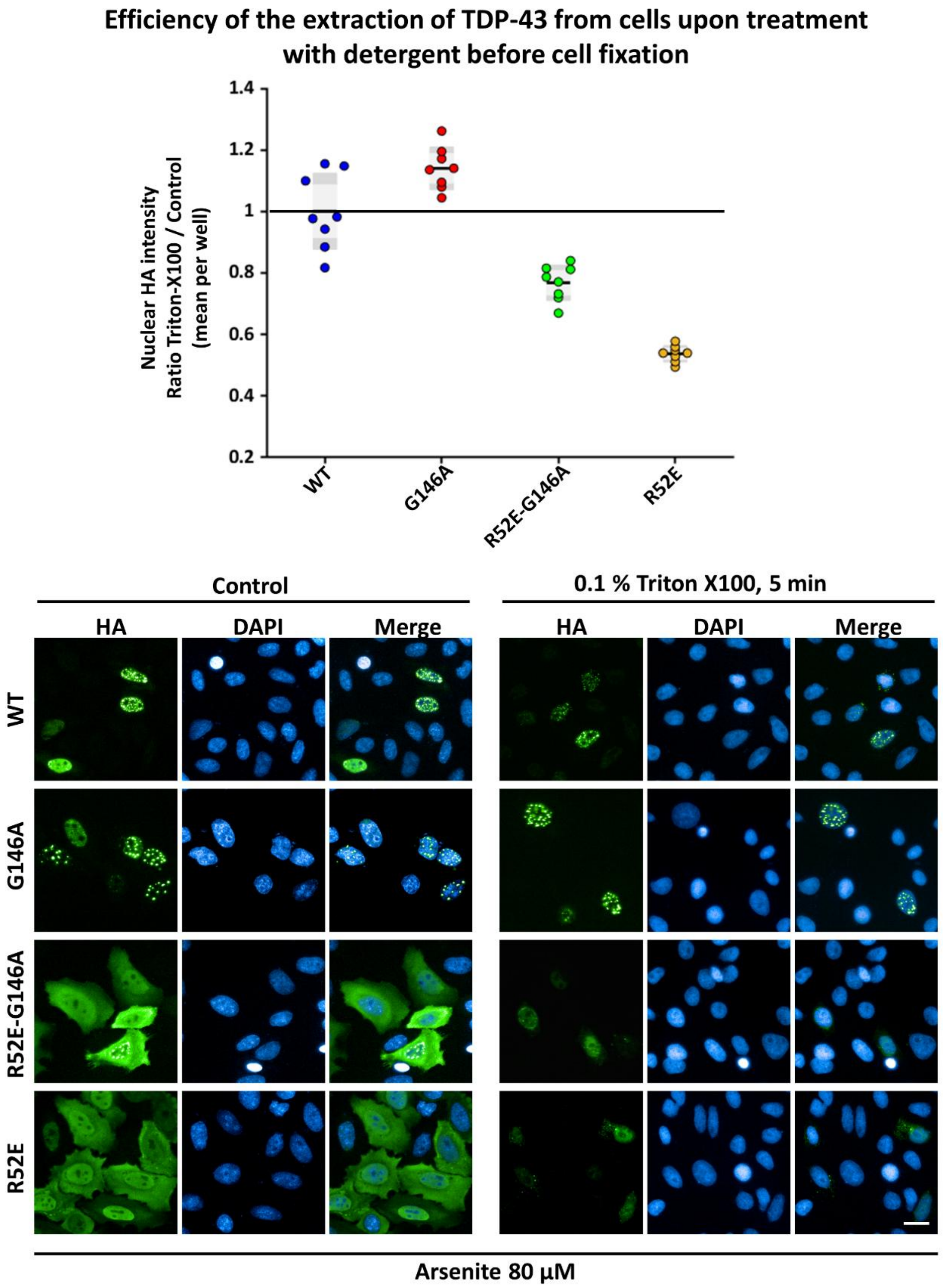

Supplementary Figure 23: Arsenite-induced TDP-43 aggregates are not dissociated by the detergent

HeLa cells expressing indicated TDP-43 mutants and treated with 80 µM arsenite were washed with PBS (control) or 0.1 % Triton X-100 for 5 min before fixation.

Upper panel: The intensity of HA-labelled TDP43 mutants in cell nuclei was measured, and the ratios of the intensities of detergent-treated to control cells are plotted. Automated measurement from cells grown in a 96-well plate (n=8 wells per condition).

Lower panel: Representative images of cells (HA-antibody). The extraction of soluble mutants (R52E and R52E-G146A) out of the nucleus and cytoplasm is observed, whereas nuclear TDP-43 aggregates remain resistant to detergent treatment. Scale bar: 20µm.

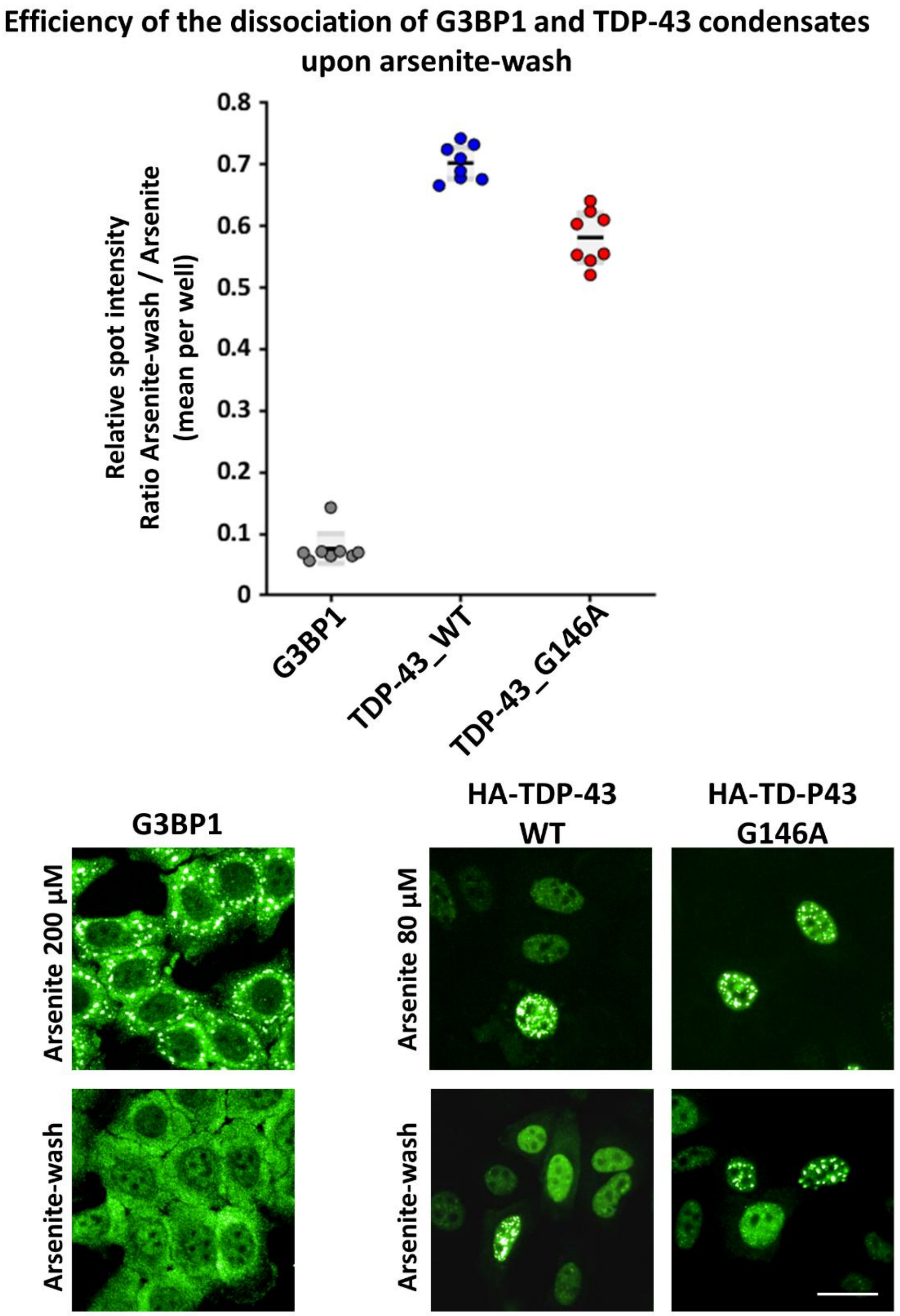

Supplementary Figure 24: Arsenite-induced TDP43 aggregates do not completely dissociate after the removal of arsenite

HeLa cells expressing the indicated TDP-43 mutants were treated with 80 µM arsenite for 1 h. Arsenite is then withdrawn, and cells are incubated for 90 min, whereas control cells are treated with arsenite without its removal. In parallel, non-transfected cells were treated with 200 µM arsenite to generate cytoplasmic stress granules, and endogenous G3BP1 was detected using specific antibodies.

Upper panel: The HA-relative intensity of nuclear aggregates versus the surrounding nucleoplasm was measured. Similarly, the G3BP1-relative intensity of stress granules in the cytoplasm was quantified. The ratios of the intensities of cells with arsenite removal to control cells are plotted. Automated measurement from cells grown in a 96-well plate (n=8 wells per condition).

Lower panel: Representative images of cells (G3BP1- and HA-antibody). Almost absolute dissociation of stress granules is observed upon the removal of arsenite, whereas nuclear TDP43 aggregates (WT and G146A) do not completely dissociate after the removal of arsenite. Scale bar: 20μm.

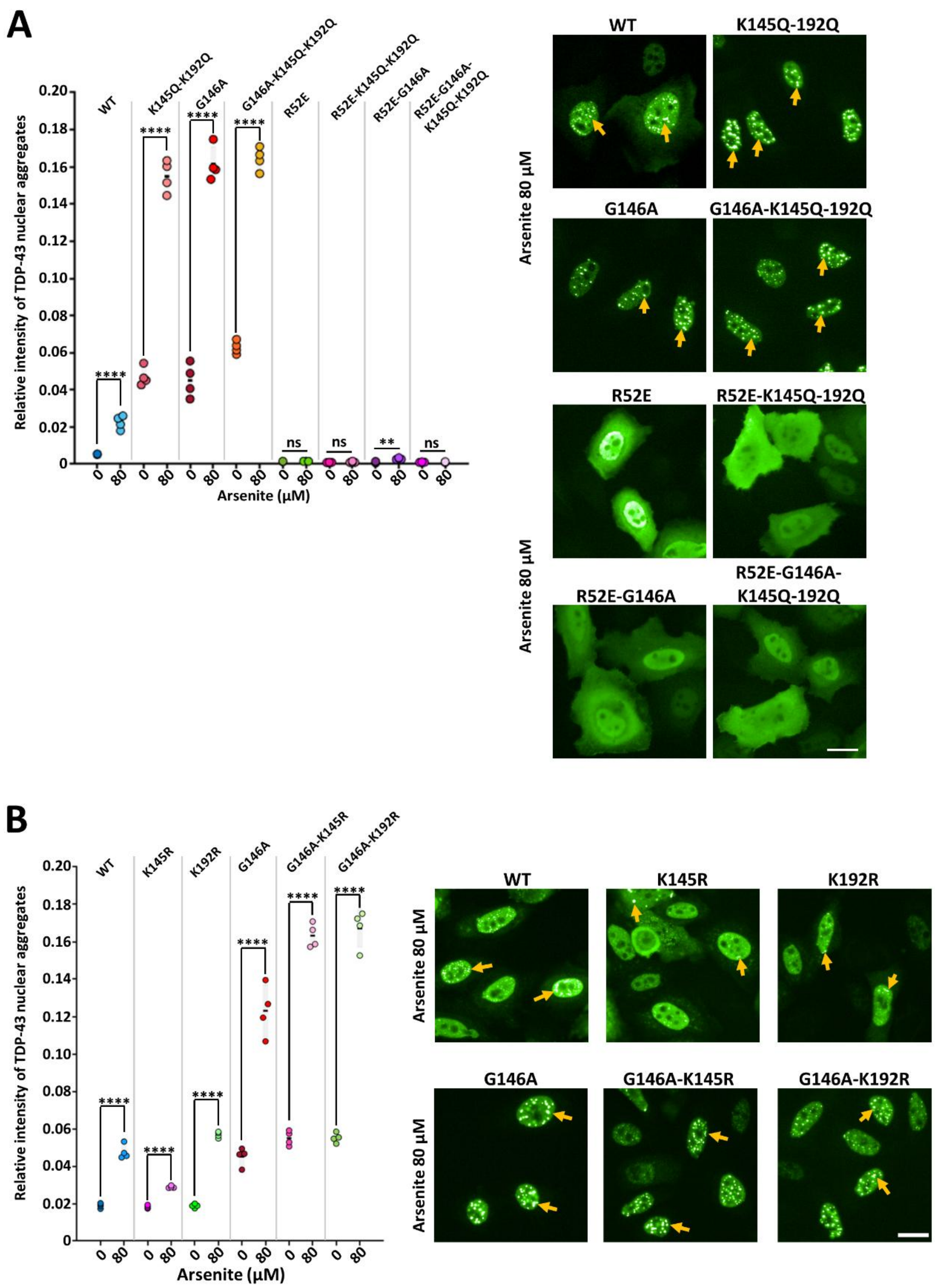

Supplementary Figure 25: Relationship between acetylation of RRMs and TDP-43 aggregation

A) Left panel: The HA-relative intensity of nuclear aggregates versus surrounding nucleoplasm was measured to score TDP-43 aggregation in HeLa cells expressing HA-tagged different TDP-43 construct under indicated arsenite concentrations. Acetylation-mimic mutations of RRM lysine residues significantly promote TDP-43 aggregation. The

asterisks indicate statistical significance with ns, non-significant, **p<0.01, ****p<0.0001, as measured by two-sided two-sample t-tests from n=4 wells (each dot represents one well). Right panel: Representative images (anti-HA). Scale bar: 20µm.

B) Left panel: The HA-relative intensity of nuclear aggregates versus surrounding nucleoplasm was measured to score TDP-43 aggregation in HeLa cells expressing HA-tagged different TDP-43 construct under indicated arsenite concentrations. Single mutations of RRM lysine residues are not sufficient to prevent TDP-43 aggregation. The asterisks indicate statistical significance with ****p<0.0001, as measured by two-sided two-sample t-tests from n=4 wells (each dot represents one well). Right panel: Representative images (anti-HA). Scale bar: 20µm.

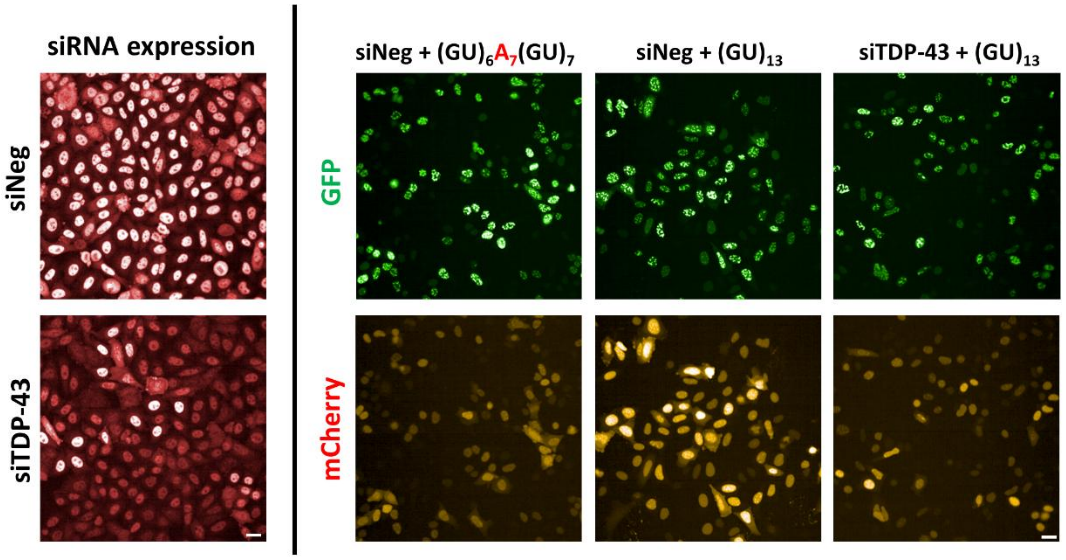

Supplementary Figure 26: Validation of the splicing reporter assays to ensure the TDP-43 dependent excision of exon 9 in the CFTR gene

Left panel: HeLa cells were treated with TDP-43 siRNA or siNEG to decrease the expression of endogenous TDP-43. Endogenous TDP-43 was detected with anti-TDP-43 antibody. The representative images clearly show the efficiency of TDP-43 siRNA. Most cells displayed a limited expression of TDP-43 in the nuclei compared to siNEG-treated cells. Scale bar: 20µm.

Right panel: Representative images of the GFP signal (representative of the expression of splicing reporter mRNA) and RFP signal (representative of the mRNA for which exon 9 has been excised). We noticed the exon 9 has been excised in cells treated with siNEG but not with siTDP-43. In addition, adding an A_7 sequence ($(GU)_6A_7(GU)_7$) in the middle of the $(GU)_{13}$ repeats, initially expressed by the splicing reporter, prevent TDP-43 to excise exon 9. Scale bar: 20µm.

Supplementary Table 1. pET plasmids used for protein production and purification

Plasmids	Encode for
pTDP-NTD_1–93	NTD fragment
pTDP-E17R_1–93	NTD fragment of TDP-43 mutant E17R
pTDP-RRM1-2_101–277	RRM1-2 fragment
pTDP-NTD-RRM1-2_1-277	NTD-RRM1-2 fragment
pTDP-E17R_1-277	NTD-RRM1-2 fragment of TDP-43 mutant E17R
pTDP-R52E_1-277	NTD-RRM1-2 fragment of TDP-43 mutant R52E

Supplementary Table 2. SAXS experimental conditions and deduced parameters from collected data. Characteristic dimensions, Rg and Dmax, and molar mass (MMcorrelation volume) were obtained from data analysis. The theoretical masses (MMsequence) were calculated from the amino acid sequence.

**Data collection parameters**				
Instrument	SWING (SOLEIL)			
Detector	Eiger4M (Dectris)			
Beam geometry	0.8 mm x 0.15 mm			
Wavelength [Å]	1.033			
q-range [$Å^{-1}$]	0.003 < q < 0.50			
Absolute scaling	Comparison with scattering from pure H_2O			
Exposure/dead time [s]	0.99 /0.01			
Loading sample concentration [µM]	1-277_WT: 60 $(GT)_{12}$:27	1-277_R52E+ 1-277_E17R: 60 $(GT)_{12}$:27	1-277_R52E: 60 $(GT)_{12}$:27	1-277_E17R: 60 $(GT)_{12}$:20
	**WT/$(GT)_{12}$**	**(R52E+E17R)/$(GT)_{12}$**	**R52E/$(GT)_{12}$**	**E17R/$(GT)_{12}$**
**Guinier analysis**				
R_g Guinier [Å]	41.70±0.28	41.38±0.38	40.73±0.49	39.22±0.32
qRg-range	0.40-1.29	0.40-1.21	0.52-1.30	0.43-1.30
**P(r) analysis**				
R_g p(r) [Å]	44.09±0.25	43.26±0.29	42.76±0.49	41.56±0.34
q-range [$Å^{-1}$]	0.010 - 0.55	0.010 - 0.55	0.010 - 0.55	0.010 - 0.55
D_{Max} [Å]	162	164	159	158
**Molecular mass determination**				
$MM_{sequence}$ [kDa]	70.5	70.5	70.5	70.5
$MM_{correlation\ volume}$ [kDa]	88.6	72.4	67.6	69.4

Loading sample concentration [µM]	1-277_WT: 60 $(GT)_6A_{12}(GT)_6$:27	1-277_R52E+ 1-277_E17R: 60 $(GT)_6A_{12}(GT)_6$:27	1-277_R52E: 60 $(GT)_6A_{12}(GT)_6$:27	1-277_E17R: 60 $(GT)_6A_{12}(GT)_6$:27
	**WT/$(GT)_6A_{12}(GT)_6$**	**(R52E+E17R) /$(GT)_6A_{12}(GT)_6$**	**R52E/$(GT)_6A_{12}(GT)_6$**	**E17R/$(GT)_6A_{12}(GT)_6$**
**Guinier analysis**				
R_g Guinier [Å]	43.39±0.24	43.76±0.34	49.03±0.41	48.23±0.57
q*Rg*-range	0.47-1.21	0.32-1.22	0.41-1.21	0.37-1.21
**P(r) analysis**				
R_g p(r) [Å]	45.19±0.17	45.92±0.26	52.20±0.44	51.26±0.63
q-range [$Å^{-1}$]	0.011 - 0.55	0.007 - 0.55	0.008 - 0.55	0.008 - 0.55
D_{Max} [Å]	166	168	203	200
**Molecular mass determination**				
$MM_{sequence}$ [kDa]	74.3	74.3	74.3	74.3
$MM_{correlation\ volume}$ [kDa]	93.9	83.6	79.3	84.1

Loading sample concentration [µM]	1-277_WT: 60 $(GU)_{12}$:20	1-277_R52E+ 1-277_E17R: 60 $(GU)_{12}$:27	1-277_R52E: 60 $(GU)_{12}$:27	1-277_E17R: 60 $(GU)_{12}$:27
	**WT/$(GU)_{12}$**	**(R52E+E17R) /$(GU)_{12}$**	**R52E/$(GU)_{12}$**	**E17R/$(GU)_{12}$**
**Guinier analysis**				
R_g Guinier [Å]	41.71±0.46	40.54±0.24	41.17±0.38	42.01±0.16
q*Rg*-range	0.30-1.22	0.30-1.20	0.71-1.28	0.46-1.21
**P(r) analysis**				
R_g p(r) [Å]	44.37±0.37	42.05±0.23	43.72±0.37	43.45±0.15
q-range [$Å^{-1}$]	0.007 - 0.55	0.007 - 0.56	0.017 - 0.56	0.011 - 0.56
D_{Max} [Å]	163	159	161	159
**Molecular mass determination**				
$MM_{sequence}$ [kDa]	70.4	70.4	70.4	70.4
$MM_{correlation\ volume}$ [kDa]	102.1	77.4	75.3	76.7

Loading sample concentration [µM]	1-277_WT: 60 $(GU)_6A_{12}(GU)_6$:20	1-277_R52E+ 1-277_E17R: 60 $(GU)_6A_{12}(GU)_6$:20	1-277_R52E: 60 $(GU)_6A_{12}(GU)_6$:20	1-277_E17R: 60 $(GU)_6A_{12}(GU)_6$:20
	**WT/$(GU)_6A_{12}(GU)_6$**	**(R52E+E17R) /$(GU)_6A_{12}(GU)_6$**	**R52E/$(GU)_6A_{12}(GU)_6$**	**E17R/$(GU)_6A_{12}(GU)_6$**
**Guinier analysis**				
R_g Guinier [Å]	39.65±0.35	41.11±0.24	48.26±0.32	45.62±0.32
q*Rg*-range	0.22-1.21	0.17-1.20	0.26-1.23	0.17-1.21
**P(r) analysis**				
R_g p(r) [Å]	40.79±0.34	42.73±0.18	51.10±0.41	48.18±0.40
q-range [$Å^{-1}$]	0.006 - 0.55	0.004 - 0.55	0.006 - 0.55	0.004 - 0.55
D_{Max} [Å]	151	156	198	195
**Molecular mass determination**				
$MM_{sequence}$ [kDa]	74.1	74.1	74.1	74.1
$MM_{correlation\ volume}$ [kDa]	85	83.7	82.0	76.7

Loading sample concentration [µM]	1-277_WT: 60 $(GT)_9$:30	1-277_R52E+ 1-277_E17R: 60 $(GT)_9$:30	1-277_R52E: 60 $(GT)_9$:30
	**WT/$(GT)_9$**	**(R52E+E17R) /$(GT)_9$**	**R52E/$(GT)_9$**
**Guinier analysis**			
R_g Guinier [Å]	37.70±0.91	35.10±0.48	35.79±0.59
q*Rg*-range	0.53-1.22	0.40-1.22	0.28-1.21
**P(r) analysis**			
R_g p(r) [Å]	39.44±0.57	36.73±0.37	37.68±0.27
q-range [$Å^{-1}$]	0.014 - 0.55	0.011 - 0.55	0.008 - 0.55
D_{Max} [Å]	139	134	132
**Molecular mass determination**			
$MM_{sequence}$ [kDa]	68.6	68.6	68.6
$MM_{correlation\ volume}$ [kDa]	56.3	45.7	42.7

Loading sample concentration [µM]	1-277_WT: 60<br>$(GT)_{19}$:10	1-277_R52E+<br>1-277_E17R: 60<br>$(GT)_{19}$:10	1-277_R52E: 60<br>$(GT)_{19}$:10
	**WT/$(GT)_{19}$**	**(R52E+E17R) /$(GT)_{19}$**	**R52E/$(GT)_{19}$**
**Guinier analysis**			
R_g Guinier [Å]	47.35±0.17	52.67±0.63	56.84±0.25
q*Rg*-range	0.39-1.30	0.26-1.20	0.31-1.27
**P(r) analysis**			
R_g p(r) [Å]	49.51±0.33	57.56±0.55	61.30±0.40
q-range [$Å^{-1}$]	0.008 - 0.55	0.005 - 0.55	0.006 - 0.55
D_{Max} [Å]	201	225	248
**Molecular mass determination**			
$MM_{sequence}$ [kDa]	106.5	106.5	106.5
$MM_{correlation\ volume}$ [kDa]	139.5	140.2	144.4

Loading sample concentration [µM]	1-277_WT: 60<br>$(GT)_{24}$:8	1-277_R52E+<br>1-277_E17R: 60<br>$(GT)_{24}$:8	1-277_R52E: 60<br>$(GT)_{24}$:8
	**WT/$(GT)_{24}$**	**(R52E+E17R) /$(GT)_{24}$**	**R52E/$(GT)_{24}$**
**Guinier analysis**			
R_g Guinier [Å]	55.44±0.72	61.26±0.83	67.64±1.43
q*Rg*-range	0.51-1.21	0.34-1.23	0.40-1.20
**P(r) analysis**			
R_g p(r) [Å]	57.29±0.63	64.96±0.49	78.76±0.92
q-range [$Å^{-1}$]	0.009 - 0.55	0.006 - 0.55	0.006 - 0.55
D_{Max} [Å]	209	231	300
**Molecular mass determination**			
$MM_{sequence}$ [kDa]	141.1	141.1	141.1
$MM_{correlation\ volume}$ [kDa]	208.7	198.5	208.1

Supplementary Table 3. Sequences of the oligonucleotides used for EMSA

Oligo Name	Sequence (5'-3')
Stem-loop $(GT)_{36}$	GAAGAAGAGAAGAAGAAG -$(GT)_{36}$- CTTCTTCTTCTCTTCTTC
Stem-loop A_{48}	GAAGAAGAGAAGAAGAAG -A_{48}- CTTCTTCTTCTCTTCTTC
Stem-loop A_{72}	GAAGAAGAGAAGAAGAAG -A_{72}- CTTCTTCTTCTCTTCTTC

Supplementary Table 4. Poly (GT) repeats binding capacity (N), apparent dissociation constant (K_D), and thermodynamic parameters for NTD-RRM1-2 fragments (a.a., 1-277) or RRM1-2 fragments (a.a., 101-277) of TDP-43, as determined by ITC. [a] and [b] correspond to apparent dissociation constants K_{D1} and K_{D2}, respectively. The thermodynamic parameters (ΔH, TΔS, ΔG) and χ^2 values were expressed in kcal/mol and (kcal/mol)^2, respectively.

Protein	Poly (GT) repeats	N	K_D (M)	ΔH	TΔS	ΔG	χ2
RRM1-2	$(GT)_6$	$0.96 \pm 1.2e^{-3}$	$40.6e^{-9} \pm 1.72e^{-9}$	$-32.8 \pm 1.0e^{-1}$	-22.4	-10.4	$5.4e^{-2}$
	$(GT)_{12}$	$0.49 \pm 2.0e^{-4}$	[a] $52.7e^{-9} \pm 4.13e^{-10}$<br>[b] $0.62e^{-9} \pm 1.32e^{-12}$	$-4.76 \pm 6.0e^{-2}$<br>$-49.4 \pm 5.2e^{-2}$	5.17<br>-36.8	-9.65<br>-12.6	$1.4e^{-2}$
1-277_WT	$(GT)_6$	$0.88 \pm 2.1e^{-3}$	$69.5e^{-9} \pm 3.37e^{-9}$	-28.0 ± 0.130	-18.2	-9.77	$5.1e^{-2}$
	$(GT)_{12}$	$0.43 \pm 2.6e^{-4}$	[a] $94.0e^{-9} \pm 3.80e^{-10}$<br>[b] $17.7e^{-6} \pm 60.0e^{-9}$	$-50.1 \pm 5.8e^{-2}$<br>-24.7 ± 0.205	40.5<br>18.3	-9.59<br>-6.48	$1.4e^{-2}$
1-277_E17R	$(GT)_6$	$0.98 \pm 1.1e^{-3}$	$79.8e^{-9} \pm 2.11e^{-9}$	$-24.3 \pm 6.1e^{-2}$	-14.6	-9.68	$9.0e^{-3}$
	$(GT)_{12}$	$0.49 \pm 5.1e^{-5}$	[a] $61.7e^{-9} \pm 3.00e^{-10}$<br>[b] $0.67e^{-9} \pm 2.09e^{-12}$	$-7.81 \pm 6.4e^{-2}$<br>$-52.1 \pm 5.6e^{-2}$	2.03<br>-39.5	-9.84<br>-12.4	$1.6e^{-2}$
1-277_R52E	$(GT)_{12}$	$0.42 \pm 7.5e^{-4}$	[a] $85.1e^{-9} \pm 5.28e^{-12}$<br>[b] $0.87e^{-9} \pm 7.11e^{-12}$	-8.37 ± 0.105<br>$-52.5 \pm 9.8e^{-2}$	1.28<br>-39.9	-9.65<br>-12.4	$4.1e^{-2}$
	$(GT)_6A_{12}(GT)_6$	$0.40 \pm 1.4e^{-3}$	$87.8e^{-9} \pm 5.01e^{-9}$	-45.8 ± 0.309	-36.2	-9.63	0.186
1-277_R52E<br>+1-277_E17R	$(GT)_{12}$	$0.40 \pm 5.8e^{-4}$	[a] $101e^{-9} \pm 9.92e^{-10}$<br>[b] $18.2e^{-6} \pm 119e^{-9}$	-52.6 ± 0.132<br>-24.4 ± 0.373	43.0<br>18.0	-9.55<br>-6.47	$5.8e^{-2}$
