# Supplementary material for: From TDP-43/RNA complex formation to disease-linked TDP-43 aggregation through a structural and cellular approach
Source: Nat Commun. 2026 Jan 21;17:1631. doi: 10.1038/s41467-026-68346-y (PMC12905282; doi:10.1038/s41467-026-68346-y)
Supplement: Supplementary file 2 — Reporting Summary [file 41467_2026_68346_MOESM2_ESM.pdf]

nature research

Corresponding author(s): Ahmed BOUHSS

Last updated by author(s): Dec 10, 2025

Reporting Summary

Nature Research wishes to improve the reproducibility of the work that we publish. This form provides structure for consistency and transparency in reporting. For further information on Nature Research policies, see Authors & Referees and the Editorial Policy Checklist.

Statistics

For all statistical analyses, confirm that the following items are present in the figure legend, table legend, main text, or Methods section.

n/a	Confirmed	
☐	☒	The exact sample size (n) for each experimental group/condition, given as a discrete number and unit of measurement
☐	☒	A statement on whether measurements were taken from distinct samples or whether the same sample was measured repeatedly
☐	☒	The statistical test(s) used AND whether they are one- or two-sided<br>*Only common tests should be described solely by name; describe more complex techniques in the Methods section.*
☒	☐	A description of all covariates tested
☒	☐	A description of any assumptions or corrections, such as tests of normality and adjustment for multiple comparisons
☐	☒	A full description of the statistical parameters including central tendency (e.g. means) or other basic estimates (e.g. regression coefficient) AND variation (e.g. standard deviation) or associated estimates of uncertainty (e.g. confidence intervals)
☐	☒	For null hypothesis testing, the test statistic (e.g. F, t, r) with confidence intervals, effect sizes, degrees of freedom and P value noted<br>*Give P values as exact values whenever suitable.*
☒	☐	For Bayesian analysis, information on the choice of priors and Markov chain Monte Carlo settings
☒	☐	For hierarchical and complex designs, identification of the appropriate level for tests and full reporting of outcomes
☒	☐	Estimates of effect sizes (e.g. Cohen's d, Pearson's r), indicating how they were calculated

Our web collection on statistics for biologists contains articles on many of the points above.

Software and code

Policy information about availability of computer code

Data collection	For microtubule bench assay, all image acquisition was carried out with Opera Phenix® Plus High Content Screening System (PerkinElmer);<br>For nuclear granules assay and Splicing reporter assay, the cell images were obtained with the Operetta CLS™ High-Content Analysis System (PerkinElmer);<br>TopSpin v3.5; FoxTrot v2.0.2; Bio-Rad Image Lab v6.0.1; MicroCal PEAQ-ITC Control Software v1.22; BIOVIA Discovery Studio Visualizer software (v24.1.0.23298).
Data analysis	The cell image analysis was performed using the PerkinElmer Harmony v5.2 software; TopSpin v3.5; CcpNmr AnalysisAssign 3.1.0; FoxTrot v2.0.2; BioXTAS RAW 2.2.1; MicroCal PEAQ-ITC Analysis Software v1.22; BIOVIA Discovery Studio Visualizer software (v24.1.0.23298).

For manuscripts utilizing custom algorithms or software that are central to the research but not yet described in published literature, software must be made available to editors/reviewers. We strongly encourage code deposition in a community repository (e.g. GitHub). See the Nature Research guidelines for submitting code & software for further information.

Data

Policy information about availability of data

All manuscripts must include a data availability statement. This statement should provide the following information, where applicable:

- Accession codes, unique identifiers, or web links for publicly available datasets
- A list of figures that have associated raw data
- A description of any restrictions on data availability

Chemical shift assignments for 15N-labelled TDP-43 protein fragments used in this study were extracted from the following entries available on the Biological Magnetic Resonance Data Bank (BMRB): 30345, 27613, 30345, 19290. The data that support the findings of this study are available from the corresponding author upon request.

Field-specific reporting

Please select the one below that is the best fit for your research. If you are not sure, read the appropriate sections before making your selection.

- [x] Life sciences
- [ ] Behavioural & social sciences
- [ ] Ecological, evolutionary & environmental sciences

For a reference copy of the document with all sections, see nature.com/documents/nr-reporting-summary-flat.pdf

Life sciences study design

All studies must disclose on these points even when the disclosure is negative.

Sample size	Microtubule bench experiments were performed as described by Maucuer et al. J Cell Sci (2018).<br>Stress granule experiments, cell cytoplasm and nucleus were detected automatically by using sofware provide with Opera Phenix® Plus High Content Screening System (PerkinElmer). The cell number chosen provides a sufficiently good statistical significance regarding the changes analyzed in this study.
Data exclusions	No data exclusion for analysis.
Replication	Attempts of replication were all successful. Experiments were repeated in two or three independent replicates, as indicated in figure legends or in the online methods to reliably support conclusions stated in the manuscript.
Randomization	No data ramdomization was performed for any of experiments cited in this study.
Blinding	No blinding was applied in this study.

Reporting for specific materials, systems and methods

We require information from authors about some types of materials, experimental systems and methods used in many studies. Here, indicate whether each material, system or method listed is relevant to your study. If you are not sure if a list item applies to your research, read the appropriate section before selecting a response.

Materials & experimental systems

n/a	Involved in the study	
[ ]	[x]	Antibodies
[ ]	[x]	Eukaryotic cell lines
[x]	[ ]	Palaeontology
[x]	[ ]	Animals and other organisms
[x]	[ ]	Human research participants
[x]	[ ]	Clinical data

Methods

n/a	Involved in the study	
[x]	[ ]	ChIP-seq
[x]	[ ]	Flow cytometry
[x]	[ ]	MRI-based neuroimaging

Antibodies

Antibodies used	anti-HA antibody (pAb Proteintech 51064-2-AP; mAb Santa Cruz Biotechnology Sc-7392); anti-TDP43 antibody (mAb ABnova H00023435-M01; pAb Proteintech 12892-1-AP); donkey anti-mouse DyLigh-488-conjugated secondary antibodies (Invitrogen, SA5-10166); rabbit polyclonal anti-G3BP1 antibodies (Sigma-Aldrich, G6046); donkey anti-rabbit DyLigh-488-conjugated secondary antibodies (Invitrogen, SA5-10038); goat anti-mouse IRDye® 680RD secondary antibodies (LI-COR, 926-68070); goat anti-rabbit IRDye® 680RD secondary antibodies (LI-COR, 926-68071).
Validation	Control consists to use cells harboring plamid expressing untagged target. All antibodies used were commercial and validated by the manufacturer.

Eukaryotic cell lines

Policy information about cell lines

Cell line source(s)	Henrietta Lacks (HeLa) cells (ATCC CCL-2).
Authentication	The cell line used in this study is authenticated by the Short Tandem Repeat (STR) method.
Mycoplasma contamination	The used cell line was tested negative for mycoplasma contamination.
Commonly misidentified lines (See ICLAC register)	The cell line used in this study is not listed in the ICLAC database.
